# 0.01% Atropine Eye Drops in Children With Myopia and Intermittent Exotropia: The AMIXT Randomized Clinical Trial

**DOI:** 10.1001/jamaophthalmol.2024.2295

**Published:** 2024-07-03

**Authors:** Zijin Wang, Tianxi Li, Xiaoxia Zuo, Tong Zhang, Lei Liu, Chenyu Zhou, Zhenhua Leng, Xuejuan Chen, Lingyan Wang, Xiaofeng Wang, Hu Liu

**Affiliations:** 1 Department of Ophthalmology, The First Affiliated Hospital with Nanjing Medical University, Nanjing, China; 2 School of Medical Technology, Jiangsu College of Nursing, Huai’an, China

## Abstract

**Importance:**

Exotropia and myopia are commonly coexistent. However, evidence is limited regarding atropine interventions for myopia control in children with myopia and intermittent exotropia (IXT).

**Objective:**

To evaluate the efficacy and safety of 0.01% atropine eye drops on myopia progression, exotropia conditions, and binocular vision in individuals with myopia and IXT.

**Design, Setting, and Participants:**

This placebo-controlled, double-masked, randomized clinical trial was conducted from December 2020 to September 2023. Children aged 6 to 12 years with basic-type IXT and myopia of −0.50 to −6.00 diopters (D) after cycloplegic refraction in both eyes were enrolled.

**Intervention:**

Participants were randomly assigned in a 2:1 ratio to 0.01% atropine or placebo eye drops administered in both eyes once at night for 12 months.

**Main Outcomes and Measures:**

The primary outcome was change in cycloplegic spherical equivalent from baseline at 1 year. Secondary outcomes included change in axial length (AL), accommodative amplitude (AA), exotropia conditions, and binocular vision at 1 year.

**Results:**

Among 323 screened participants, 300 children (mean [SD] age, 9.1 [1.6] years; 152 male [50.7%]) were included in this study. A total of 200 children (66.7%) were in the atropine group, and 100 (33.3%) were in the placebo group. At 1 year, the 0.01% atropine group had slower spherical equivalent progression (−0.51 D vs −0.75 D; difference = 0.24 D; 95% CI, 0.11-0.37 D; *P* < .001) and AL elongation (0.31 mm vs 0.42 mm; difference = −0.11 mm; 95% CI, −0.17 to −0.06 mm; *P* < .001) than the placebo group. The mean AA change was −3.06 D vs 0.12 D (difference = −3.18 D; 95% CI, −3.92 to −2.44 D; *P* < .001) in the atropine and placebo groups, respectively. The 0.01% atropine group had a decrease in near magnitude of exodeviation whereas the placebo group had an increase (−1.25 prism diopters [PD] vs 0.74 PD; difference = −1.99 PD; 95% CI, −3.79 to −0.19 PD; *P* = .03). In the atropine vs placebo group, respectively, the incidence of study drug-related photophobia was 6.0% (12 of 200 participants) vs 8.0% (8 of 100 participants; difference = −2.0%; 95% CI, −9.4% to 3.7%; *P* = .51) and for blurred near vision was 6.0% (12 of 200 participants) vs 7.0% (7 of 100 participants) (difference = −1.0%; 95% CI, −8.2% to 4.5%; *P* = .74).

**Conclusions and Relevance:**

The findings of this randomized clinical trial support use of 0.01% atropine eye drops, although compromising AA to some extent, for slowing myopia progression without interfering with exotropia conditions or binocular vision in children with myopia and IXT.

**Trial Registration:**

Chinese Clinical Trial Registry Identifier: ChiCTR2000039827

## Key Points

**Question:**

Are 0.01% atropine eye drops effective and safe for children with myopia and intermittent exotropia?

**Findings:**

In this randomized clinical trial including 300 participants, 0.01% atropine eye drops led to slower progression in cycloplegic spherical equivalent measurement and slower elongation in axial length than placebo at 1 year, with no substantial adverse effects on exotropia conditions or binocular vision.

**Meaning:**

These results suggest that 0.01% atropine eye drops are effective and safe in slowing myopia progression without interfering with exotropia conditions or binocular vision in children with myopia and intermittent exotropia.

## Teaser

This parallel-design, double-masked, randomized clinical trial investigates the effectiveness and safety of atropine, 0.01%, eye drops for children with myopia and intermittent exotropia.

## Introduction

Intermittent exotropia (IXT), characterized by an intermittent outward deviation of 1 or both eyes, is one of the most common types of strabismus, particularly in Asian countries.^1^ The prevalence of IXT was reported to be 1.0% in the US^1^ and 4.7% in Asia.^2^

Exotropia and myopia are commonly coexistent. The myopia prevalence rate in populations with exotropia has been reported to reach as high as 57.7% in 12-year-old Australian children, much higher than that in children without strabismus (12.3%).^3^ A cross-sectional study of children aged 6 to 72 months in the Multi-Ethnic Pediatric Eye Disease Study (MEPEDS) in southern California and the Baltimore Pediatric Eye Disease Study (BPEDS) in Maryland revealed a higher prevalence rate of myopia in children with exotropia (12.7%) than that in children without exotropia (4.6%).^4^ Myopia has been identified as a risk factor for concomitant exotropia. It is thought that myopia is associated with a decreased demand for accommodation and, hence, lower convergence and a predisposition for developing exotropia.^5^ Population-based studies support this viewpoint, with myopia found to increase the risk of IXT development by 5.2-fold.^6^ On the other hand, IXT has been regarded as a risk factor for myopia onset and progression. It was reported that more than 90% of patients with IXT would develop myopia by 20 years of age, which was higher than that in populations without strabismus.^7^ In patients with IXT, additional accommodative convergence is required to maintain binocular vision and ocular alignment, which might increase accommodative loads and, hence, myopia progression.^8^ Alternatively, it was speculated that increased convergence demand (via convergence accommodation/convergence), rather than accommodation, contributes to the myopia development in IXT.^9^

Due to divisive definitions of exotropia deterioration, the natural history of IXT remains controversial. In the past decade, results from several multicenter clinical studies, especially those from the Pediatric Eye Disease Investigator Group (PEDIG), suggested observation as a preferable option for patients with IXT who had good exotropia control, stable magnitude of exodeviation, and mild psychological pressure.^10,11^ Thus, increased attention and further investigation seem warranted in the management of myopia progression in children with IXT, prompting this evaluation of low-concentration atropine in children with IXT, as previous clinical trials of atropine for myopia control excluded children with strabismus.^12,13,14,15,16,17^ The current 2-year, placebo-controlled, randomized clinical trial included evaluation of the effects of 0.01% atropine eye drops on accommodative changes, exotropia conditions, and binocular vision in children with myopia and IXT (AMIXT) for which we report the 1-year primary results.

## Methods

### Study Design and Study Population

This trial was conducted from December 2020 to September 2023 at the First Affiliated Hospital with Nanjing Medical University, Nanjing, China. Ethics committee approval was obtained from the institutional review board of the First Affiliated Hospital with Nanjing Medical University. This double-masked, single-center, randomized clinical trial enrolled children aged 6 to 12 years with basic-type IXT and myopia of −0.50 to −6.00 diopters (D) after cycloplegic refraction in both eyes. All participants were of Han ethnicity due to geographical distribution. Informed written consent was obtained from parents or guardians, and additional informed written consent was obtained from participants 8 years and older. Detailed inclusion and exclusion criteria can be found in the trial protocol (Supplement 1). Participants received free examinations without a stipend. The trial was registered with the Chinese Clinical Trial Registry and followed the Consolidated Standards of Reporting Trials (CONSORT) reporting guidelines. All study procedures followed the tenets of the Declaration of Helsinki.

### Randomization and Masking

Eligible participants were randomly assigned 2:1 to receive 0.01% atropine or placebo eye drops by use of a block randomization scheme with a fixed block size of 6. The random allocation sequence was generated using randomized block methods in the R Statistical Package, version 4.0.0 (R Foundation for Statistical Computing) by a statistician who was not involved in participant recruitment or data collection. Allocation of treatment drugs was performed by 1 unmasked investigator (L.W.) who was not responsible for data collection or data analysis using opaque, sealed, and sequentially numbered envelopes that contain randomized numbers. Participants and investigators who evaluated the outcome measurements were masked to study allocation.

### Study Intervention and Termination

Participants received 0.01% atropine or placebo eye drops (1% hydroxypropyl methylcellulose) in both eyes once at night for 12 months. All eye drops were prepared in the same monodose package of 0.4-mL unit concentration without preservatives, with the same solvent (1% hydroxypropyl methylcellulose) and pH. The 0.01% atropine or placebo eye drops were all prepared by Shenyang Xingqi Pharmaceutical Co in Shenyang, China. The quality control certificates were provided for each batch of eye drops by the manufacturer.

The intervention was discontinued if the guardian or participant requested to withdraw or if the participant presented with severe allergic response or aggravation of IXT fulfilling the deterioration criteria for 2 consecutive follow-up visits. IXT deterioration criteria were met by either development of constant exotropia of 10 prism diopters (PD) or greater at distance and near or a decrease in near stereoacuity of at least 2 octaves.^10^ When the deterioration criteria were first met, a retest was performed after a 10-minute break to confirm or refute the result. The participants experiencing deterioration for 2 consecutive follow-up visits were requested to cease the intervention and then underwent a further 2-month observation period. If participants still fulfilled the deterioration criteria, surgery was recommended.

### Procedures

The participants underwent a regular assessment every 2 months (±2 weeks) during the first 6 months, followed by assessments every 3 months (±2 weeks) during the last 6 months.

Axial length (AL), best-corrected visual acuity (BCVA), near vision, accommodative amplitude (AA), photopic and mesopic pupil size, distant and near magnitude of exodeviation, distant and near control,^18^ distant^19^ and near stereoacuity, near point of convergence (NPC), accommodative convergence/accommodation (AC/A) ratio, fusional vergence amplitude (FVA), and intraocular pressure (IOP) were evaluated at each follow-up visit, whereas cycloplegic spherical equivalent and corneal endothelial cell density (ECD) were measured every 6 months. Spherical equivalent was calculated as spherical power plus one-half of the cylinder power. The magnitude of exodeviation was measured with prisms and the alternate-cover test after 1 hour of monocular occlusion, which was thought to approach the largest exodeviation in an individual with IXT. All the procedures were performed by trained ophthalmologists and optometrists, who were masked to the treatment assignment. A detailed description of study procedures and devices can be found in the trial protocol (Supplement 1).

Participants were inquired whether photophobia existed at baseline because some patients with IXT may have this symptom.^20^ Administration compliance of atropine or placebo eye drops was evaluated based on the eye drop diary, number of empty monodose containers returned, and inquiries on times of missed administrations at each visit. Individuals who used 75% or more of the prescribed medication were considered to have good compliance. Daily outdoor hours and self-reported symptoms related to allergy, blurred near vision, photophobia, or any discomfort were inquired at each visit.

### Outcome Measures

The primary outcome was cycloplegic spherical equivalent change from baseline at 1 year. Secondary outcomes included change from baseline in AL, monocular function (BCVA, near vision, AA and photopic/mesopic pupil size), exotropia conditions (distant/near magnitude of exodeviation and distant/near exotropia control), binocular vision (distant/near stereoacuity, NPC, AC/A ratio, and FVA), and safety parameters (IOP and ECD) at 1 year.

### Sample Size and Statistical Analysis

The sample size was determined using PASS, version 15 (NCSS) assuming: (1) an annual reduction in spherical equivalent of 0.59 D in the 0.01% atropine group and 0.81 D in the placebo group according to results of the Low-Concentration Atropine for Myopia Progression (LAMP) study,^15^ (2) the common SD was assumed to be 0.57 D in each group, and (3) random assignment to the 0.01% atropine group and placebo group was 2:1. We calculated that the trial needed 160 participants in the 0.01% atropine group and 80 participants in the placebo group to provide 80% power (at 5% type I error rate) for detecting a clinically relevant difference of 0.22 D in spherical equivalent progression at 12 months. To account for an expected dropout rate of 20% over the 12-month follow-up period, the total number of participants for enrollment would need to be 300 or more.

### Statistical Analysis

Statistical analyses followed the intention-to-treat principle. Data from children who were randomly assigned to treatment and completed at least 1 follow-up visit were included in the analysis. Descriptive results were presented as mean (SD) for continuous measures and number (percentage) for categorical measures. Eye-specific outcome measures were compared between treatment groups using generalized linear models, and generalized estimating equations (GEEs) were used to account for intereye correlation. For comparing primary outcome and secondary outcomes between treatment groups, the longitudinal analysis was performed using GEE models with an autoregressive correlation structure. The GEE models analyzed data from all participants including those who withdrew from the trial during the follow-up, assuming that the missing data were completely at random, which were checked by comparing baseline characteristics between participants with vs without completion of the 12-month follow-up visit. For the change in spherical equivalent and AL, the prespecified subgroup analyses were performed for age (≤8 years vs >8 years), sex (male vs female), baseline spherical equivalent (≤−2 D vs >−2 D), baseline AL (≤24 mm vs >24 mm), distant magnitude of exodeviation at baseline (≤20 PD vs >20 PD), and daily outdoor hours (≤2 hours vs >2 hours). The differences in treatment effect between subgroups were evaluated based on the test of interaction between treatment group and subgroup. A 2-sided *P* value <.05 was considered statistically significant for the primary outcome. All secondary outcomes were considered for hypothesis generation as there was no adjustment for multiplicity. All statistical analyses were performed using SPSS, version 21.0 (IBM Corp), and R, version 4.3.1 (R Foundation for Statistical Computing).

## Results

**Figure 1.**
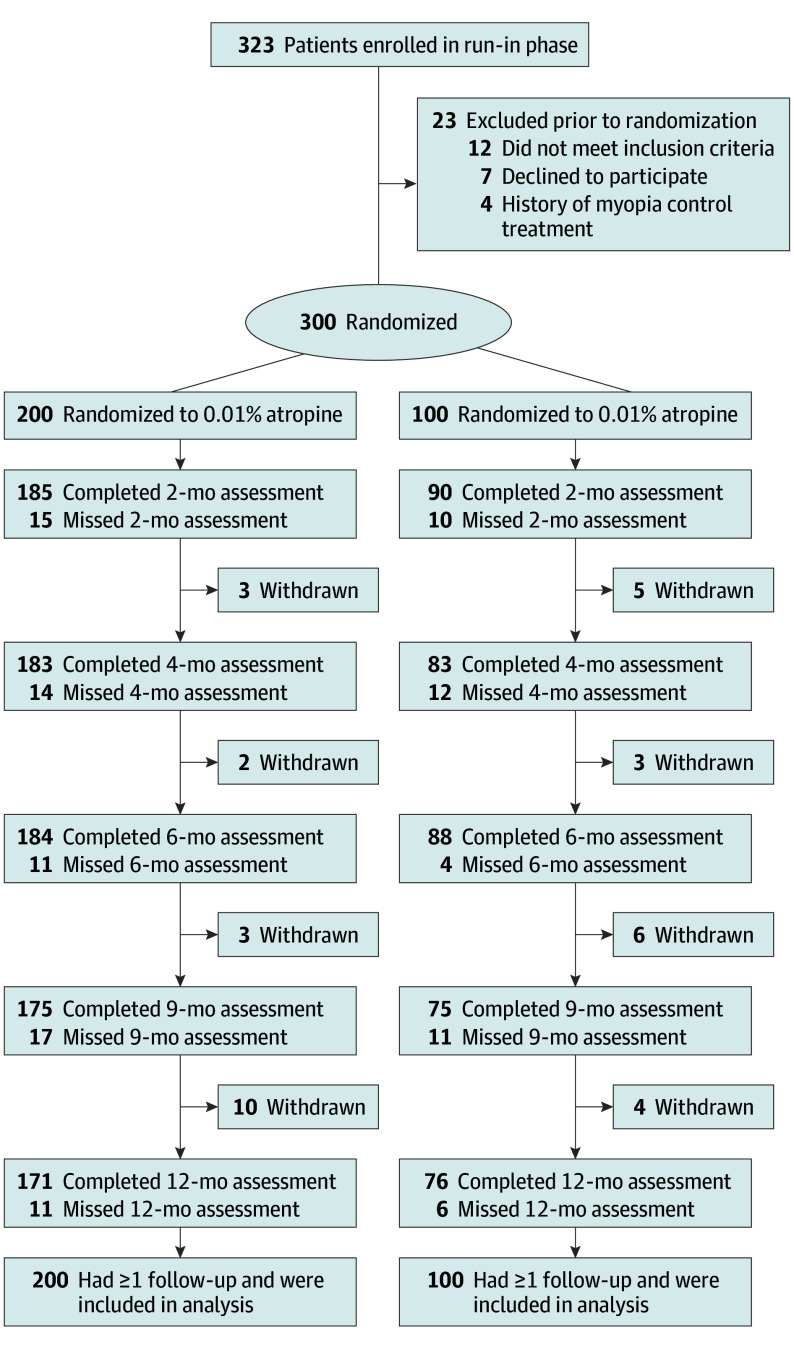
Flow of Participants Through Trial

Among 323 study participants assessed for eligibility, 300 children (mean [SD] age, 9.1 [1.6] years; 148 female [49.3%]; 152 male [50.7%]; Han ethnicity [100%]) were eligible and enrolled. A total of 200 children (66.7%) were randomly assigned to the 0.01% atropine group, and 100 (33.3%) were randomly assigned to the placebo group. At 12 months, follow-up was completed by 171 of 200 children (85.5%) in the 0.01% atropine group and 76 of 100 children (76.0%) in the placebo group. All participants received treatment and completed at least 1 follow-up visit and, thus, were included in the statistical analysis (Figure 1). Good treatment compliance at 1 year was 97.1% (166 of 171 children) and 93.4% (71 of 76 children) in the atropine and placebo groups, respectively. Daily outdoor time was not different between treatment groups (2.28 [0.05] hours vs 2.14 [0.07] hours; difference = 0.14 hours; 95% CI, −0.03 to 0.31 hours; *P* = .11). Baseline characteristics of participants, available in Table 1, appeared similar across groups and baseline characteristics appeared similar between participants who completed (n = 247) or did not complete (n = 53) the 12-month follow-up (eTable 1 in Supplement 2).

**Table 1.** Baseline Characteristics of Study Participants

Characteristics	0.01% Atropine (n = 200)	Placebo (n = 100)
Age, mean (SD), y	9.14 (1.58)	9.10 (1.65)
Sex, No. (%)		
Female	98 (49.0)	50 (50.0)
Male	102 (51.0)	50 (50.0)
Cycloplegic spherical equivalent, mean (SD), D	−2.32 (1.29)	−2.27 (1.31)
Axial length, mean (SD), mm	24.58 (0.83)	24.42 (0.88)
Distant best-corrected visual acuity, mean (SD), logMAR	−0.06 (0.06)	−0.05 (0.07)
Near vision, mean (SD), logMAR	0.08 (0.09)	0.08 (0.11)
Accommodative amplitude, mean (SD), D	17.19 (5.26)	16.98 (5.11)
Photopic pupil size, mean (SD), mm	2.55 (0.71)	2.41 (0.72)
Mesopic pupil size, mean (SD), mm	6.38 (0.82)	6.34 (0.80)
Distant magnitude of exodeviation, mean (SD), PD	20.79 (6.93)	21.20 (7.35)
Near magnitude of exodeviation, mean (SD), PD	27.13 (7.37)	26.43 (7.63)
Distant exotropia control, mean (SD)	2.23 (0.65)	2.18 (0.54)
Distant exotropia control, No. (%)		
0	0	0
1	0	1 (1.0)
2	174 (87.0)	85 (85.0)
3	10 (5.0)	10 (10.0)
4	12 (6.0)	3 (3.0)
5	4 (2.0)	1 (1.0)
Near exotropia control, mean (SD)	0.68 (0.89)	0.71 (1.04)
Near exotropia control, No. (%)		
0	110 (55.0)	59 (59.0)
1	53 (26.5)	19 (19.0)
2	30 (15.0)	18 (18.0)
3	5 (2.5)	1 (1.0)
4	2 (1.0)	2 (2.0)
5	0 (0.0)	1 (1.0)
Distant stereoacuity, mean (SD), log arcsecs	3.08 (0.25)	3.10 (0.24)
Near stereoacuity, mean (SD), log arcsecs	1.71 (0.19)	1.70 (0.21)
Near point of convergence, mean (SD), cm	4.19 (2.37)	4.47 (1.79)
AC/A mean (SD),	1.80 (1.31)	1.79 (1.33)
Fusional vergence amplitude,^a^ mean (SD), degrees	19.55 (8.68)	18.93 (8.35)
Fusional convergence, mean (SD), degrees	13.40 (7.41)	13.18 (7.22)
Fusional divergence, mean (SD), degrees	6.16 (2.80)	5.75 (2.53)
Intraocular pressure, mean (SD), mm Hg	16.11 (2.80)	15.87 (2.76)
Endothelial cell density, mean (SD), cells/mm^2^	3425.41 (261.96)	3399.67 (266.57)

Abbreviations: AC/A, accommodative convergence/accommodation; D, diopter; PD, prism diopter.

^a^ Fusional vergence amplitude was derived from 193 participants in the 0.01% atropine group and 97 participants in the placebo group, whereas the other participants had no fusional function.

### Change in Cycloplegic Spherical Equivalent

**Figure 2.**
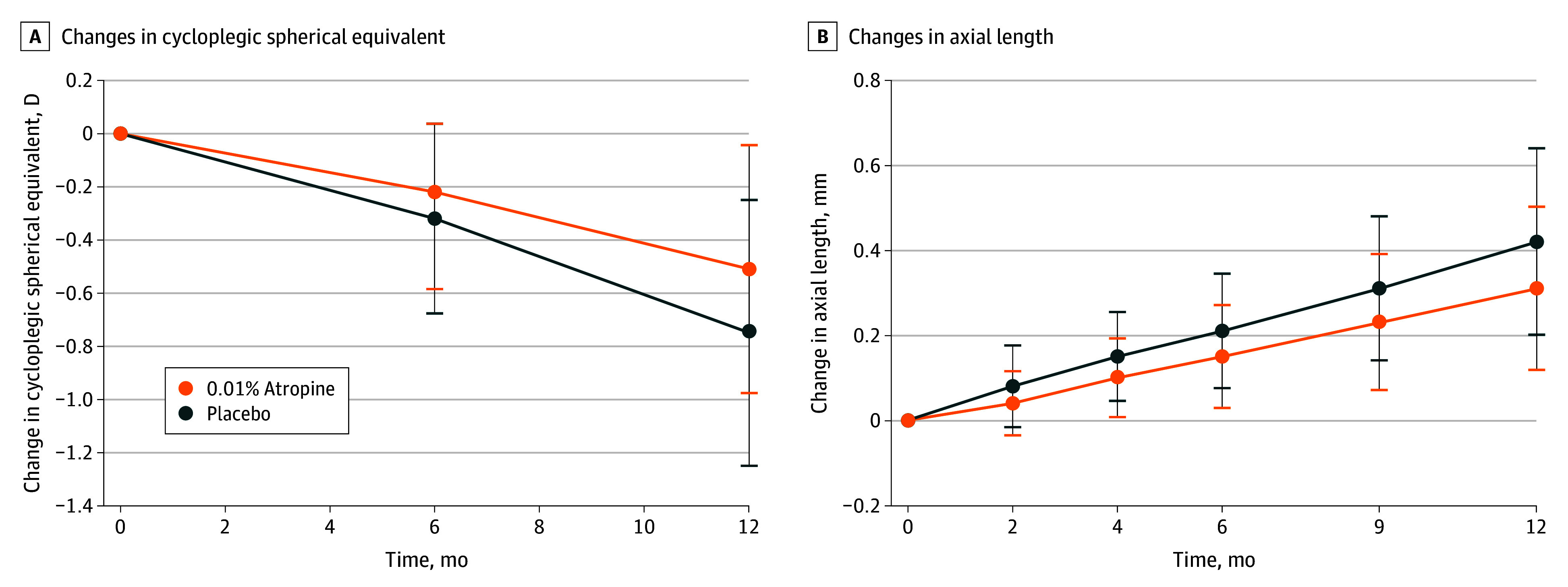
Changes in Cycloplegic Spherical Equivalent and Axial Length Across Time by Treatment Groups A, Cycloplegic spherical equivalent. B, Axial length. Data are presented with mean with SD over 1 year. D indicates diopter.

**Figure 3.**
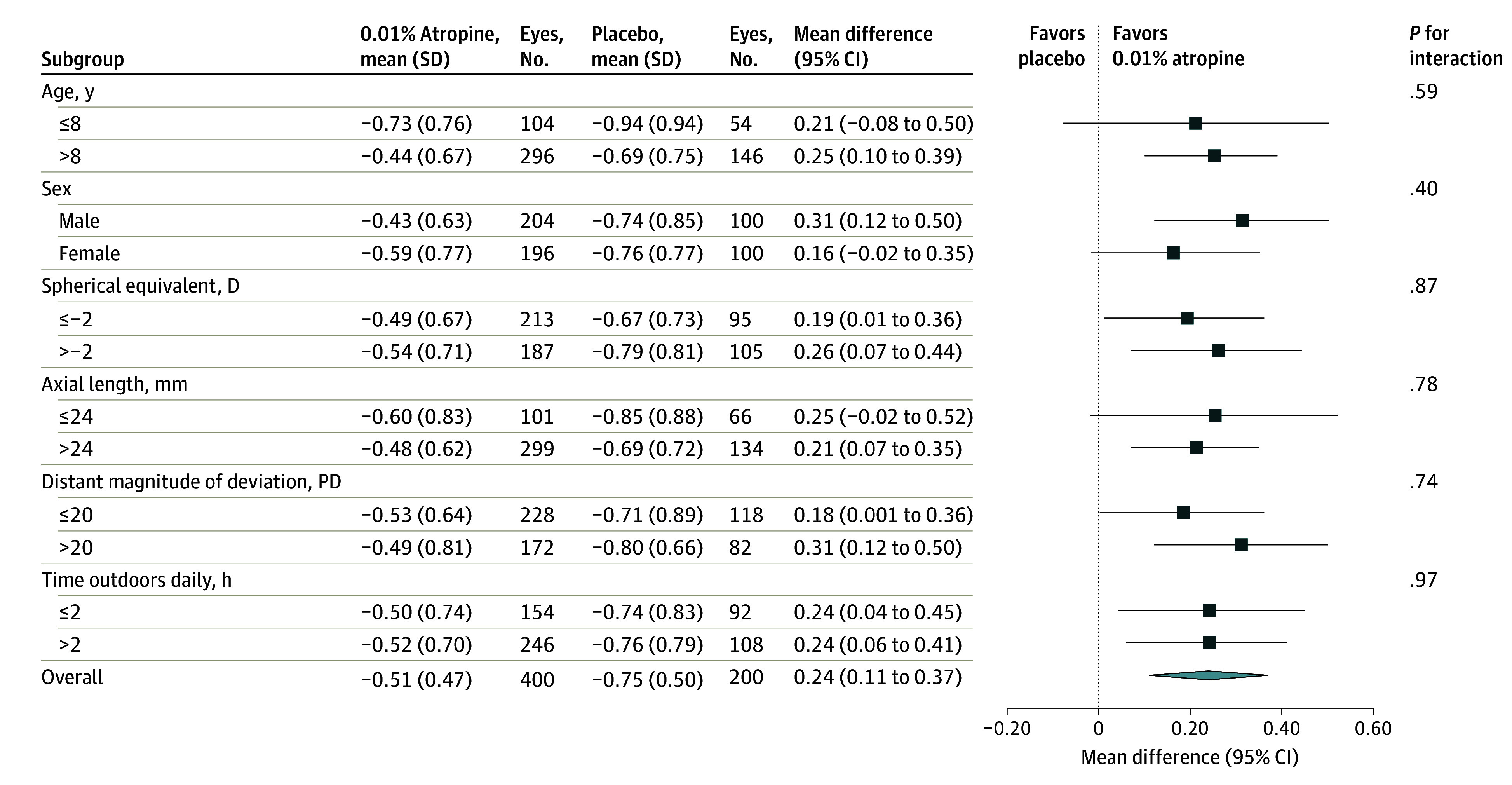
Subgroup Analyses for Change in Cycloplegic Spherical Equivalent at 1 Year The interaction between treatment groups and subgroups was evaluated using multivariable generalized estimating equations models. D indicates diopter; PD, prism diopter.

At 1 year, the mean (SD) change from baseline in cycloplegic spherical equivalent (Table 2) was −0.51 (0.47) D in the atropine group and −0.75 (0.50) D in the placebo group (difference = 0.24 D; 95% CI, 0.11-0.37 D; *P* < .001). Mean difference between treatment groups appeared to gradually increase with time (Figure 2A). The proportion of eyes with spherical equivalent progression of less than 0.50 D at 1 year (eFigure 1 in Supplement 2) was 49.4% (169 of 342 eyes) in the atropine group and 30.3% (46 of 152 eyes) in the placebo group (difference = 19.2%; 95% CI, 9.8%-27.7%; *P* = .006). The proportion of eyes with spherical equivalent progression of 1.0 D or more was 19.3% (66 of 342 eyes) and 36.2% (55 of 152 eyes) in the atropine and placebo groups, respectively (difference = −16.9%; 95% CI, −25.7% to −8.4%; *P* = .008). Subgroup analyses did not appear to show an interaction on the treatment effect (Figure 3).

**Table 2.** Change From Baseline in Ocular Outcome Measures at 1 Year by Treatment Groups

Outcome measures at 1 y	Change, mean (SD)^a^		Mean between-group difference (95% CI)	*P* value
	0.01% Atropine (n = 200)	Placebo (n = 100)		
Cycloplegic spherical equivalent, D	−0.51 (0.47)	−0.75 (0.50)	0.24 (0.11 to 0.37)	<.001
Axial length, mm	0.31 (0.19)	0.42 (0.22)	−0.11 (−0.17 to −0.06)	<.001
Distant best-corrected visual acuity, logMAR	−0.01 (0.06)	0.00 (0.05)	−0.01 (−0.03 to 0.00)	.08
Near vision, logMAR	−0.02 (0.08)	−0.01 (0.09)	−0.01 (−0.03 to 0.01)	.39
Accommodative amplitude, D	−3.06 (2.96)	0.12 (2.63)	−3.18 (−3.92 to −2.44)	<.001
Photopic pupil size, mm	0.65 (0.60)	−0.01 (0.67)	0.67 (0.49 to 0.84)	<.001
Mesopic pupil size, mm	0.55 (0.56)	0.04 (0.45)	0.51 (0.37 to 0.64)	<.001
Distant magnitude of exodeviation, PD	−1.04 (3.89)	−0.02 (4.62)	−1.03 (−2.22 to 0.17)	.09
Near magnitude of exodeviation, PD	−1.25 (6.04)	0.74 (6.93)	−1.99 (−3.79 to −0.19)	.03
Distant exotropia control	−0.05 (0.99)	0.00 (0.87)	−0.05 (−0.30 to 0.20)	.69
Near exotropia control	−0.12 (0.85)	−0.05 (0.90)	−0.08 (−0.32 to 0.16)	.53
Distant stereoacuity, log arcsec	−0.01 (0.33)	−0.01 (0.28)	0.00 (−0.08 to 0.08)	.94
Near stereoacuity, log arcsec	−0.04 (0.15)	−0.02 (0.18)	−0.01 (−0.06 to 0.04)	.64
Near point of convergence, cm	0.53 (1.92)	0.62 (1.88)	−0.09 (−0.60 to 0.42)	.74
Accommodative convergence/accommodation	0.23 (1.72)	0.36 (1.64)	−0.13 (−0.58 to 0.33)	.59
Fusional vergence amplitude, degrees	−1.42 (9.30)	−0.64 (8.64)	−0.78 (−3.17 to 1.61)	.52
Fusional convergence amplitude, degrees	−1.33 (8.37)	−0.63 (7.76)	−0.70 (−2.84 to 1.45)	.52
Fusional divergence amplitude, degrees	−0.13 (2.62)	−0.01 (2.53)	−0.12 (−0.57 to 0.81)	.73
Intraocular pressure, mm Hg	−0.75 (2.53)	−0.45 (2.49)	−0.31 (−0.98 to 0.37)	.79
Corneal endothelial cell density, cells/mm^2^	−31.73 (96.18)	−51.81 (108.46)	20.09 (−8.24 to 48.42)	.17

Abbreviations: D, diopter; PD, prism diopter.

^a^ The changes in ophthalmic parameters at follow-up visits were analyzed using generalized estimating equation models.

### Change in AL

The mean (SD) increase in AL (Table 2) was 0.31 (0.19) mm in the atropine group and 0.42 (0.22) mm in the placebo group at 1 year (difference = −0.11 mm; 95% CI, −0.17 to −0.06 mm; *P* < .001). During 1-year follow-up, the mean AL difference between the 2 groups appeared to gradually increase with time (Figure 2B). In subgroup analyses (eFigure 2 in Supplement 2), no treatment interaction was noted.

### Change in Monocular Function

As shown in Table 2, the atropine and placebo groups showed no differences for change in BCVA or near vision at 1 year. The mean (SD) AA change (Table 2) was −3.06 (2.96) D in the atropine group and 0.12 (2.63) D in the placebo group (difference = −3.18 D; 95% CI, −3.92 to −2.44 D; *P* < .001) at 1 year. The decrease in AA in the atropine group began at 2 months and remained stable over time (eFigure 3 in Supplement 2). The atropine group had more increase in photopic pupil size (mean [SD], 0.65 [0.60] mm vs −0.01 [0.67] mm; difference = 0.67 mm; 95% CI, 0.49-0.84 mm; *P* < .001) and mesopic pupil size (mean [SD], 0.55 [0.56] mm vs 0.04 [0.45] mm; difference = 0.51 mm; 95% CI, 0.37-0.64; *P* < .001) than in the placebo group (Table 2). The difference appeared stable starting at 2 months (eFigure 3 in Supplement 2).

### Change in Exotropia Conditions

In the atropine vs placebo group, respectively, the mean (SD) change was −1.04 (3.89) PD vs −0.02 (4.62) PD (difference = −1.03 PD; 95% CI, −2.22 to 0.17 PD; *P* = .09) in distant magnitude of exodeviation, −0.05 (0.99) vs 0 (0.87) (difference = −0.05; 95% CI, −0.30 to 0.20; *P* = .69) in distant exotropia control, and −0.12 (0.85) vs −0.05 (0.90; difference = −0.08; 95% CI, −0.32 to 0.16; *P* = .53) in near exotropia control at 1 year (Table 2). Near magnitude of exodeviation fluctuated over time (eFigure 4 in Supplement 2), with mean (SD) change from baseline at 1 year of −1.25 (6.04) PD vs 0.74 (6.93) PD (difference = −1.99 PD; 95% CI, −3.79 to −0.19 PD; *P* = .03) (Table 2). The means and SDs of these parameters over time are shown in eFigure 5 in Supplement 2. Regarding deterioration, none of the participants met the motor criteria for 2 consecutive visits, but a total of 4 participants in the atropine group and 2 participants in the placebo group met stereoacuity deterioration criteria temporarily (eTable 2 in Supplement 2). All recovered at the following visit.

### Change in Binocular Vision

At 1 year, 0.01% atropine eye drops did not show an effect on binocular vision (Table 2). All these measures appeared stable over time (eFigure 6 in Supplement 2).

### Change in Safety Parameters

Change in IOP (mean [SD], −0.75 [2.53] mm Hg vs −0.45 [2.49] mm Hg; difference = −0.31 mm Hg; 95% CI, −0.98 to 0.37 mm Hg; *P* = .79) and ECD (mean [SD], −31.73 [96.18] cells/mm^2^ vs −51.81 [108.46] cells/mm^2^; difference = 20.09 cells/mm^2^; 95% CI, −8.24 to 48.42 cells/mm^2^; *P* = .17) appeared similar between treatment groups at 1 year, with ECD decreasing over time in both treatment groups (eFigure 7 in Supplement 2).

### Adverse Effects

The rate of photophobia at baseline was 16.5% (33 of 200 participants) in the atropine group and 15.0% (15 of 100 participants) in the placebo group (difference = 1.5%; 95% CI, −7.9% to 9.6%; *P* = .74). In the atropine vs placebo groups, respectively, study drug–related photophobia was 6.0% (12 of 200 participants) vs 8.0% (8 of 100 participants; difference = −2.0%; 95% CI, −9.4% to 3.7%; *P* = .51) and for blurred near vision was 6.0% (12 of 200 participants) vs 7.0% (7 of 100 participants; difference = −1.0%; 95% CI, −8.2% to 4.5%; *P* = .74). Although 11 participants in the atropine group and 5 participants in the placebo group were diagnosed with allergic conjunctivitis, none were judged associated with study drugs.

## Discussion

The AMIXT randomized clinical trial evaluated the efficacy and safety of 0.01% atropine eye drops on myopia progression, exotropia conditions, binocular vision, monocular function, and safety parameters in individuals with myopia and IXT at 1 year. The 0.01% atropine group had slower myopia progression in both spherical equivalent measurement and AL among children with myopia and IXT. The 0.01% atropine group did not appear to have aggravated exotropia conditions, in terms of magnitude of exodeviation and exotropia control. The 0.01% atropine group did not appear to have worse binocular vision, supported by assessment of distant stereoacuity, near stereoacuity, NPC, AC/A, and FVA. In addition, the use of 0.01% atropine appeared safe with a mild decrease in AA and a mild increase in pupil size; stability of BCVA, near vision, IOP, and ECD; and low incidence of adverse events.

In this trial, we found that administration of 0.01% atropine eye drops for 1 year reduced myopia progression by a mean of 0.24 D in spherical equivalent and reduced AL elongation by a mean of 0.11 mm, appearing similar to treatment effects found in the population of individuals with myopia and without strabismus.^15,21,22,23,24,25,26,27,28^ Mean differences between the 0.01% atropine and control groups ranged from 0.10 D to 0.96 D, whereas the mean difference between groups in annual AL change in these studies ranged from 0.05 mm to 0.48 mm.^15,23,24,25,26,27,28^ The proportion of rapid myopia progression (≥1 D in 1 year) in this trial was 19.3% in the atropine group and 36.2% in the placebo group, which was generally consistent with results reported in previous studies such as 27.8% vs 37.1%,^15^ 13.2% vs 34.9%,^23^ and 20.3% vs 35.6^24^ in 0.01% atropine and placebo groups, respectively, in the population of individuals without strabismus.

Prespecified exploratory subgroup analyses in this study showed a treatment effect consistent across all subgroups. Several previous studies reported an age-dependent effect of low-concentration atropine on treatment response^29,30,31^; although, this was not supported in 1 study.^32^ Female sex was reported to be a risk factor for natural myopia progression,^29,33^ and a risk factor for poor response to low-concentration atropine in a previous study.^34^ Another previous study^35^ found that rapid AL elongation after atropine administration was related significantly to shorter AL at baseline. Previous studies regarding associations of baseline spherical equivalent with treatment response yielded inconsistent conclusions.^29,30,32,36^

Previous randomized clinical trials have reported that 0.01% atropine administration led to annual reductions in AA ranging from 0.26 to 4.4 D.^14,15,17^ In our study, the mean decrease in AA in the 0.01% atropine group was 3.06 D, appearing at the 2-month visit and stabilized thereafter. Initially, we were concerned that the decrease in AA might reduce accommodative convergence and cause aggravation of IXT. However, a case series study^37^ reported the development of convergence-excess consecutive esotropia in 3 patients who underwent IXT surgery and attributed this result to preoperative and postoperative use of 0.01% atropine eye drops. The attribution came from the finding that the obvious postoperative esotropia at near resolved within 3 weeks after atropine suspension. It was suggested that the anticholinergic drugs’ incomplete paresis of peripheral accommodation could induce greater central accommodative effort and, consequently, greater accommodative convergence, leading to the development of esotropia.^38^ Our results showed no definitive decrease in the near magnitude of exodeviation with 0.01% atropine and no differences for changes in the distant magnitude of exodeviation and distant or near exotropia control between groups, supporting the safety of 0.01% atropine in this trial. Adopting the definition of deterioration from the PEDIG study,^11^ we had 4 participants who met deterioration criteria (all for stereoacuity) at 1 visit only, improving spontaneously. None of our trial participants met the deterioration criteria for 2 consecutive visits in 1 year.

We noted no IOP or ECD safety issues. In a previous laboratory study,^39^ atropine was suggested to induce time-dependent reduction of ECD. However, a meta-analysis involving the comparison of ECD change between the orthokeratology plus 0.01% atropine group and the orthokeratology group revealed no difference.^40^ Decreasing ECD over time in our study has been noted in numerous previous studies.^40,41^

### Limitations

This study has some limitations. The single-center study design may limit the generalization of these findings. Another limitation is the lack of correction for multiple comparisons from many secondary outcomes; thus, some of the significant differences can be due to chance. The high frequency of follow-up visits along with the COVID-19 lockdown led to some missing data. Nonetheless, we delivered study drugs to the participants who had missed or delayed visits. We also kept close contact with participants, presumably improving treatment compliance and reducing the dropout rate. We applied GEE for longitudinal analysis assuming missing data completely at random,^42^ which was suggested by the similarity in their baseline characteristics in participants who completed 12-month follow-up and those who did not complete 12-month follow-up. Although this study supported the use of 0.01% atropine for the first year, it is uncertain whether efficacy and safety will be maintained in the second year; this will be reported when participants complete 2 years of follow-up. Also of note, the SD of baseline distant exotropia control in this trial is small compared with some other published data,^10,43^ and most participants had relatively good control at baseline. It is possible that our participants did not represent the full clinical spectrum of IXT because those with poorer or worsening control may be more easily noticed, and their guardians were more likely to ask for surgery.

## Conclusions

In summary, this placebo-controlled, double-masked, randomized clinical trial established that 0.01% atropine eye drops, although compromising AA to some extent, appeared effective and safe in slowing myopia progression without interfering with exotropia conditions or binocular vision in children with myopia and IXT.

## Notes

Supplement 1.
Trial Protocol.

Supplement 2. **eTable 1.** Baseline Characteristics in Participants With and Without Completion of the 12-Month Follow-Up Visit **eTable 2.** Deterioration Definitions and Outcomes **eFigure 1.** Frequency for Levels of Change From Baseline in Spherical Equivalent at 1 Year by Treatment Groups **eFigure 2.** Subgroup Analyses for Change in Axial Length at 1 Year **eFigure 3.** Change in Monocular Function by Treatment Groups Over Time Including Best-Corrected Visual Acuity, Near Vision, Accommodative Amplitude, Photopic Pupil Size, and Mesopic Pupil Size **eFigure 4.** Change in Exotropia Conditions by Treatment Groups Over Time Including Distant Magnitude of Exodeviation, Near Magnitude of Exodeviation, Distant Exotropia Control, and Near Exotropia Control **eFigure 5.** Means and Standard Deviations of Exotropia Conditions by Treatment Groups Over Time Including Distant Magnitude of Exodeviation, Near Magnitude of Exodeviation, Distant Exotropia Control, and Near Exotropia Control **eFigure 6.** Change in Binocular Vision by Treatment Groups Over Time Including Distant Stereoacuity, Near Stereoacuity, Near Point of Convergence, Accommodative Convergence/Accommodation, Fusional Vergence Amplitude, Fusional Convergence, and Fusional Divergence **eFigure 7.** Change in Safety Measures by Treatment Group Over Time Including Intraocular Pressure and Corneal Endothelial Cell Density

Supplement 3.
Data Sharing Statement.
