# Supplementary material for: 0.01% Atropine Eye Drops in Children With Myopia and Intermittent Exotropia: The AMIXT Randomized Clinical Trial
Source: JAMA Ophthalmol. 2024 Jul 3;142(8):722–30. doi: 10.1001/jamaophthalmol.2024.2295 (PMC11223046; doi:10.1001/jamaophthalmol.2024.2295)
Supplement: Supplement 1. — Trial Protocol. [file jamaophthalmol-e242295-s001.pdf]

0.01% Atropine Eyedrops in Children with Myopia and Intermittent Exotropia (AMIXT): A Randomized Clinical Trial Protocol

Version: V3.1, 15 October 2020

Primary registry and trial identifying number: ChiCTR2000039827

Original Protocol: 24 November 2019

Protocol Amendment (1): 05 December 2019

Protocol Amendment (2): 09 April 2020

Protocol Amendment (3): 12 July 2020

Protocol Amendment (4): 25 August 2020

Protocol Amendment (5): 15 October 2020

Contents

1. Investigators

2. Synopsis

3. Introduction

4. Objectives

5. Material and methods

- 5.1 Study design
- 5.2 Setting
- 5.3 Scheme
- 5.4 Participants adherence
- 5.5 Eligibility (Inclusion criteria, Exclusion criteria)
- 5.6 Sample size
- 5.7 Randomization and masking
- 5.8 Study intervention and termination
- 5.9 Outcomes
  - 5.9.1 Primary outcome
  - 5.9.2 Secondary outcomes
- 5.10 Study visit schedule and procedure
- 5.11 Clinical examinations

6. Data collection and management

7. Statistical analysis

8. Monitoring

9. Adverse event

10. Ethics and governance

11. Participant confidentiality

12. Data access and dissemination policy

13. Declaration of interests

1. Investigators

Principal investigator: Hu Liu[1]

Co-investigator: Zijin Wang[1]

1. Department of Ophthalmology, The First Affiliated Hospital with Nanjing Medical University, Nanjing, China.

2. Synopsis

Data category	Information
Primary registry and trial identifying number	ChiCTR2000039827
Date of registration in primary registry	November 11, 2020
Secondary identifying numbers	N/A
Source(s) of monetary or material support	N/A
Primary sponsor	N/A
Secondary sponsor(s)	N/A
Contact for public queries	Hu Liu<br>+86 (25) 68303165<br>The First Affiliated Hospital with Nanjing Medical University, 300 Guangzhou Road, Nanjing 210029, China; liuhu@njmu.edu.cn
Contact for scientific queries	Zijin Wang<br>+86 (25) 68303165<br>The First Affiliated Hospital with Nanjing Medical University, 300 Guangzhou Road, Nanjing 210029, China; Drzijinwang@163.com
Public title	0.01% atropine eyedrops in children with myopia and intermittent exotropia
Scientific title	0.01% atropine eyedrops in children with myopia and intermittent exotropia (AMIXT): a randomized clinical trial
Countries of recruitment	China
Health condition(s) or problem(s) studied	myopia, intermittent exotropia
Intervention(s)	Active comparator: 0.01% atropine sulfate

	Placebo comparator: 1% hydroxypropyl methylcellulose
Key inclusion and exclusion criteria	**Inclusion criteria**<br>1) age 6-12 years old;<br>2) basic-type IXT meeting all the following criteria:<br>(a.) intermittent or constant exotropia at distance (6 m), and either an IXT or exophoria at near (33 cm);<br>(b.) exodeviation magnitude of ≥10 prism diopters (PD) at distance measured by the prism and alternate cover test (PACT);<br>(c.) near/distance deviation difference of ≤10 PD;<br>3) myopia ≤-0.5 diopters (D) and >-6 D in both eyes based on cycloplegic spherical equivalent (SE);<br>4) distant best-corrected visual acuity (BCVA) of ≤0.2 logarithms of the minimum angle of resolution (logMAR) in each eye as measured using Early Treatment for Diabetic Retinopathy Study (ETDRS) charts.<br>**Exclusion criteria**<br>1) having known contraindications or sensitivity to atropine;<br>2) with serious health problems or cognitive disability affecting follow-up or ability to cooperate with examinations;<br>3) having astigmatism with cycloplegic cylinder ≥2 D in either eye;<br>4) participating in other interventional trials;<br>5) having undergone any myopia control lenses including orthokeratology, rigid gas-permeable contact lenses, progressive addition spectacle lenses, or other lenses, except for single-vision eyeglasses;<br>6) with previous use of atropine within 6 months;<br>7) having active ocular inflammation in either eye;<br>8) with peripheral anterior chamber depth <1/3 corneal thickness;<br>9) having intraocular pressure (IOP) >21 mmHg in either eye;<br>10) diagnosed of ocular diseases or having clinically significant abnormal findings in the ocular examinations;<br>11) unwilling to follow up for 2 years.
Study type	Interventional<br>Allocation: 2:1 randomized

	Intervention model: parallel assignment<br>Masking: double-blind (subject, investigator)<br>Primary purpose: treatment
Target sample size	300
Primary outcome(s)	Change in cycloplegic SE from baseline at 1 year.
Key secondary outcomes	1) Cycloplegic SE change from baseline at 2 years.<br>2) Change from baseline in axial length, monocular function (BCVA, near vision, accommodative amplitude [AA] and photopic/mesopic pupil size), exotropia conditions (distant/near magnitude of exodeviation and distant/near exotropia control), binocular vision (distant/near stereoacuity, near point of convergence [NPC], accommodative convergence/accommodation [AC/A] ratio, and fusional vergence amplitude [FVA]) and safety parameters (intraocular pressure [IOP] and corneal endothelial cell density [ECD]) at 1 year and 2 years.<br>3) Change in choroidal parameters and higher-order aberrations.

3. Introduction

Intermittent exotropia (IXT) is one of the most common types of strabismus, particularly in Asian countries,[1] which is characterized by an intermittent outward deviation of one or both eyes. The prevalence of IXT was reported to be 1.0% in the United States[1] and up to be 3.2% in Asia.[2]

Exotropia and myopia are commonly coexistent. The myopia prevalence rate in exotropia population was reported to reach 57.7%, much higher than that in children without strabismus (12.3%) in 12-year-old Australian children.[3] A cross-sectional study of 9970 children aged 6 to 72 months in the Multi-Ethnic Pediatric Eye Disease Study (MEPEDS) in southern California and the Baltimore Pediatric Eye Disease Study (BPEDS) in Maryland revealed

a higher prevalence rate of myopia in exotropia (12.7%) than that in children without exotropia (4.6%).[4] The coexistence of myopia and IXT is commonly observed, but their relationship remains controversial. On one hand, myopia has been identified as a risk factor for concomitant exotropia. It is thought that myopia is associated with a decreased demand for accommodation, and hence lower convergence and a predisposition for developing exotropia.[5] Subsequent population-based studies support this viewpoint, with myopia found to increase the risk of IXT development by 5.2 times.[6] On the other hand, IXT has been regarded as a risk factor for myopia onset and progression. It was reported that more than 90% of IXT patients would develop myopia by 20 years old, which was much higher than that in non-strabismus population.[7] In patients with IXT, additional accommodative convergence is required to maintain binocular vision and ocular alignment due to insufficient fusional convergence, which might give rise to increased accommodative loads and hence myopia progression.[8] Alternately, it was proposed that increased convergence demand (via convergence accommodation/convergence) rather than accommodation contributes to myopia development in IXT.[9] Whatever the exact mechanism is, myopia and IXT are closely related. Due to divisive definitions of exotropia deterioration, the natural history of IXT remains controversial. In the past decade, several prospective and multicenter clinical studies, especially the Pediatric Eye Disease Investigator Group (PEDIG), recommended observation as a preferable option for patients of IXT with good

exotropia control, stable magnitude of exodeviation, and mild psychological pressure.[10] Thus, increased attention and further investigation are warranted in the management of myopia progression in children with IXT.

Atropine is an emerging pharmacological method for controlling myopia progression.[11] The Atropine for the Treatment of Myopia (ATOM) study proposed 0.01% atropine as a comparably effective and less visually disruptive concentration than 0.1% and 0.5% atropine for myopia control.[12,13] Since then, 0.01% atropine has been increasingly used in clinical practice for myopia control in Asia in recent years. The Low-Concentration Atropine for Myopia Progression (LAMP) study compared the efficacy of various low concentrations of atropine in slowing myopia progression and axial elongation, with 0.05% identified as the optimal concentration.[14] While the safety and efficacy of low-concentration atropine have been repeatedly validated in non-strabismus population, there lacks evidence regarding that in population with myopia and IXT. As all the relative clinical trials before excluded participants with strabismus, whether the accommodative changes induced by atropine would impose any influence on exotropia conditions or binocular vision remains unknown. For safety, we will start with a relatively low concentration (0.01%).

4. Objectives

4.1 Primary objective

To evaluate the efficacy of 0.01% atropine eyedrops in slowing myopia progression in children with myopia and IXT.

4.2 Secondary objective

4.2.1 Key secondary objective

To determine the effect of 0.01% atropine eyedrops on exotropia conditions in children with myopia and IXT.

4.2.2 Other secondary objective

To determine the effect of 0.01% atropine eyedrops on monocular function, binocular vision, and safety parameters in children with myopia and IXT.

4.3 Exploratory objective

To explore the mechanism of 0.01% atropine eyedrops in controlling myopia progression.

5. Materials and methods

5.1 Study design

The study will be a 2-year, single-center, randomized, double-masked, and placebo-controlled trial, with a 2:1 allocation to intervention (0.01% atropine eyedrop) or placebo (1% hydroxypropyl methylcellulose).

5.2 Setting

The enrollment and follow-up of all trial participants will be at the Department of Ophthalmology, the First Affiliated Hospital with Nanjing Medical University, Nanjing, Jiangsu, China.

5.3 Scheme

Figure 1. Study flow diagram.

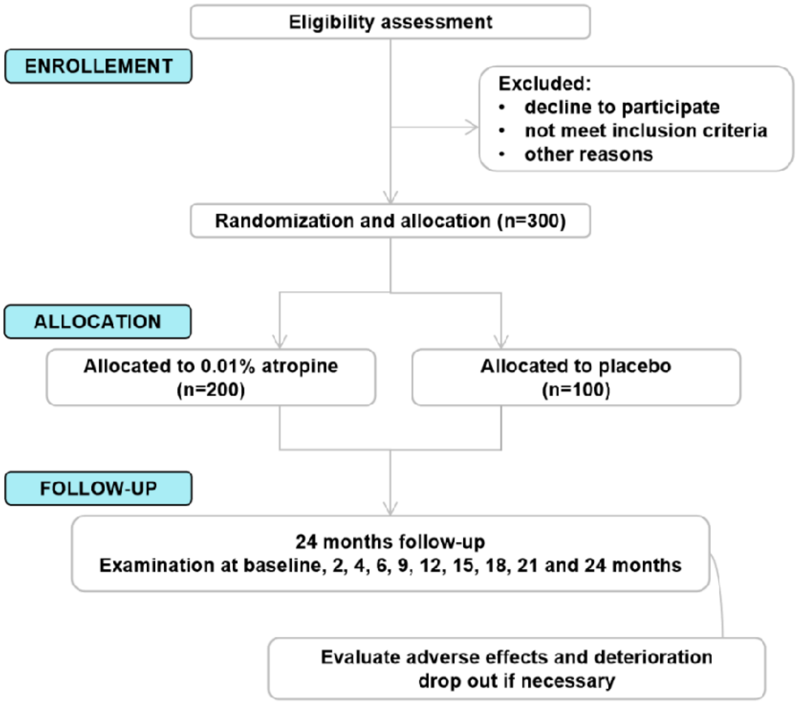

5.4 Participants adherence

Compliance of administration of atropine or placebo eyedrops is evaluated based on the eyedrop diary, number of empty mono-dose containers returned, and inquiries on times of missed administrations at each visit. Individuals who used 75% or more of the prescribed medication is considered to have good compliance.

5.5 Eligibility

Inclusion criteria

1) age 6-12 years old;

2) basic-type IXT meeting all the following criteria:

(a.) intermittent or constant exotropia at distance (6 m), and either an IXT or exophoria at near (33 cm);

(b.) exodeviation magnitude of ≥10 prism diopters (PD) at distance measured by the prism and alternate cover test (PACT);

(c.) near/distance deviation difference of ≤10 PD;

3) myopia ≤-0.5 diopters (D) and >-6 D in both eyes based on cycloplegic spherical equivalent (SE);

4) distant best-corrected visual acuity (BCVA) of ≤0.2 logarithms of the minimum angle of resolution (logMAR) in each eye as measured using Early Treatment for Diabetic Retinopathy Study (ETDRS) charts.

Exclusion criteria

1) having known contraindications or sensitivity to atropine;

2) with serious health problems or cognitive disability affecting follow-up or ability to cooperate with examinations;

3) having astigmatism with cycloplegic cylinder ≥2 D in either eye;

4) participating in other interventional trials;

5) having undergone any myopia control lenses including orthokeratology, rigid gas-permeable contact lenses, progressive addition spectacle lenses, or other lenses, except for single-vision eyeglasses;

6) with previous use of atropine within 6 months;

7) having active ocular inflammation in either eye;

8) with peripheral anterior chamber depth <1/3 corneal thickness;

9) having intraocular pressure (IOP) >21 mmHg in either eye;

10) diagnosed of ocular diseases or having clinically significant abnormal findings in the ocular examinations;

11) unwilling to follow up for 2 years.

5.6 Sample size

The sample size is determined using PASS 15 (NCSS, Utah) assuming: (1) the annual reduction of SE as 0.59 D in the 0.01% atropine group and 0.81 D in the control group according to results of the LAMP study; [14] (2) the common standard deviation (SD) is assumed as 0.57 D in each group; (3) randomization to 0.01% atropine group and placebo control group is 2:1. The trial needs 160 participants in the 0.01% atropine group and 80 participants in the control group to provide 80% power (at 5% type I error rate) for detecting a clinically relevant difference of 0.22 D in SE progression at 12 months. To account for an expected drop-out rate of 20% over the 12 months follow-up period, the total number of participants for enrollment is 300 participants (200 in 0.01% atropine group and 100 in placebo control group).

5.7 Randomization and masking

Eligible participants will be 2:1 randomized to receive 0.01% atropine or placebo eyedrops by use of a block randomization scheme with a fixed block size of six. Randomization will be completed by one unmasked investigator using sealed envelopes that contain randomized numbers. The random allocation sequence is generated using randomized block methods in the

Statistical Package for the Social Sciences (R Foundation for Statistical Computing. https://www.R-project.org/) by a statistician who is not involved in participant recruitment or data collection. Other investigators, participants, and the statistician will be masked to study allocation.

5.8 Study intervention and termination

All eyedrops (0.01% atropine sulfate, 1% hydroxypropyl methylcellulose) will be prepared in the same single-dose package without preservative, with the same solvent (1% hydroxypropyl methylcellulose). Participants will receive one drop in each eye once per night.

The single-dose containers of eyedrops will be stored in the area only accessible to the investigator who is not masked to treatment. The eyedrops should be stored at room temperature (15-25℃).

Participants and their guardians will be instructed on the administration of eyedrops after recruitment. A 2- or 3-month dosage of eyedrops will be provided at each visit after finishing all the ocular examinations, and all the empty eyedrop containers will be returned at the next visit, to calculate the compliance rate.

The intervention will be discontinued if the guardian or participant requests to withdraw, or participants has severe allergic response, or aggravation of IXT fulfilling the deterioration criteria for two consecutive follow-ups. IXT deterioration criteria will be met by either development of constant exotropia ≥10 PD at distance and near or presenting with a decrease

in near stereoacuity of at least 2 octaves.[10] When the deterioration criteria are first met, a retest will be performed after a 10-minute break to confirm or refute the result. The participants experiencing deterioration for 2 consecutive follow-ups will be requested to cease the intervention, and then underwent a further two-month observation period. If participants still fulfill the deterioration criteria, surgery will be recommended.

5.9 Outcomes

5.9.1 Primary outcome

The primary outcome is cycloplegic SE change from baseline at 1 year.

5.9.2 Secondary outcomes

1) Cycloplegic SE change from baseline at 2 years.

2) Change from baseline in axial length, monocular function (BCVA, near vision, accommodative amplitude [AA] and photopic/mesopic pupil size), exotropia conditions (distant/near magnitude of exodeviation and distant/near exotropia control), binocular vision (distant/near stereoacuity, near point of convergence [NPC], accommodative convergence/accommodation [AC/A] ratio, and fusional vergence amplitude [FVA]) and safety parameters (intraocular pressure [IOP] and corneal endothelial cell density [ECD]) at 1 year and 2 years.

3) Change in choroidal parameters and higher-order aberrations.

5.10 Study visit schedule and procedures

The participants will undergo a regular assessment every 2 months (± 2 weeks)

during the first 6 months, followed by assessments every 3 months (± 2 weeks) during the subsequent 18 months. Table 1 shows the visit schedule and study procedures.

Table 1. Study procedures at baseline and each follow-up visit.

Items	Baseline	2 months	4 months	6 months	9 months	12 months	15 months	18 months	21 months	24 months
Inclusion/exclusion criteria assessment	√									
Informed consent	√									
Enrollment and randomization	√									
Training and instruction for instillation of eyedrops	√									
Noncycloplegic refraction (Topcon KR-8900; retinoscopy)	√	√	√	√	√	√	√	√	√	√
Best-corrected distance visual acuity (logMAR, ETDRS charts)	√	√	√	√	√	√	√	√	√	√
Near vision (logMAR, illiterate “E” charts)	√	√	√	√	√	√	√	√	√	√
Slit lamp biomicroscopy	√	√	√	√	√	√	√	√	√	√
Non-mydriatic fundus examination (FundusVue)	√	√	√	√	√	√	√	√	√	√
Biometry before cycloplegia (Zeiss IOLMaster 700)	√	√	√	√	√	√	√	√	√	√
Accommodative amplitude (Near point rule)	√	√	√	√	√	√	√	√	√	√
Mesopic/photopic pupil size (OPD-Scan III)	√	√	√	√	√	√	√	√	√	√
Intraocular pressure (iCare)	√	√	√	√	√	√	√	√	√	√
Corneal endothelial cell density	√			√		√		√		√

(Topcon SP-1P)										
Distant/Near magnitude of exodeviation (Prism and alternate cover test)	√	√	√	√	√	√	√	√	√	√
Distant/near exotropia control (Office-based scale)	√	√	√	√	√	√	√	√	√	√
Distant/Near stereoacuity (Random Dots Distant Test; Randot Preschool Stereoacuity test)	√	√	√	√	√	√	√	√	√	√
Near point of convergence (Near point rule)	√	√	√	√	√	√	√	√	√	√
Fusional vergence amplitude (Synoptophore)	√	√	√	√	√	√	√	√	√	√
Accommodative convergence/accommodation (Far gradient method)	√	√	√	√	√	√	√	√	√	√
Dominant eye	√	√	√	√	√	√	√	√	√	√
Wavefront aberrometry (Tracey iTrace)	√	√	√	√	√	√	√	√	√	√
Choroidal structural and vascular component (OCT/OCTA, Optovue)	√	√	√	√	√	√	√	√	√	√
Cycloplegic refraction (Topcon KR-8900)	√			√		√		√		√
Mydriatic fundus examination (Volk Digital Wide Field Lens)	√			√		√		√		√
Dispensation of study medication/dosing compliance diary and symptom inquiry	√	√	√	√	√	√	√	√	√	√

Daily activity questionnaire	√	√	√	√	√	√	√	√	√	√

5.11 Clinical examinations

1) Noncycloplegic refraction

- Topcon KR-8900 (Topcon Corp, Tokyo, Japan) and manual refraction

2) BCVA

- ETDRS charts (Good-Lite Company, Illinois, USA)
- 4 meters
- With the manual refraction and trial frames

3) Near vision

- Illiterate “E” charts (Precision Vision, Sussex Vision Intl. Ltd, West Sussex, UK)
- 40 cm
- With the manual refraction and trial frames

4) Slit lamp biomicroscopy

- To evaluate the eyelid, eyelashes, conjunctiva, cornea, anterior chamber, iris, pupil, lens, and anterior vitreous

5) Non-mydriatic fundus examination

- FundusVue (Crystalvue Medical Corporation, Taiwan, China)
- To provide color retinal image

6) Biometry before cycloplegia

- Zeiss IOL Master 700 (Carl Zeiss Meditec Inc, Dublin, CA)
- To obtain ocular parameters, including AL

7) Accommodative amplitude (AA)

- Near point rule (rod with a moveable target and metric markings)
- Monocularly
- With the manual refraction and trial frames

- The near point of accommodation is determined when the participant reports that the letters become blurred, and clarity can not be regained, when the target is moved towards the participant's eyes.

8) Photopic and mesopic pupil size

- OPD-Scan III (Nidek, Gamagori, Japan)
- Average of at least 5 consecutive measures

9) Intraocular pressure (IOP)

- ICare rebound tonometer (Icare Finland Oy, Helsinki, Finland)
- Average of 3 IOP values

10) Corneal endothelial cell density

- Topcon SP-1P (Topcon Corp, Tokyo, Japan)

11) Distant/near magnitude of exodeviation

- PACT
- Accommodative targets for fixation at distance (6 m) and near (33 cm)
- Measure after 1 hour of monocular occlusion using a patch.

12) Distant and near control

- IXT office control scale ranging from 0 (phoria, best control) to 5 (constant exotropia, worst control) at both distance and near fixation.[15]
- Intermittent Exotropia Control Scale:

  5 = Constant exotropia

  4 = Exotropia > 50% of the exam before dissociation

  3 = Exotropia < 50% of the exam before dissociation

  2 = No exotropia unless dissociated, recovers in > 5 seconds

  1 = No exotropia unless dissociated, recovers in 1–5 seconds

  0 = No exotropia unless dissociated, recovers in < 1 second (phoria)

Levels 5 to 3 are assessed during an initial 30-second period of observation. Levels 2 to 0 are graded as the worst of three rapidly successive trials. An occluder is placed over the right eye for 10 seconds and then removed, measuring the length of time it takes for fusion to become re-established. The left eye is then occluded for a 10-second period and the time to re-fusion is similarly measured. A third trial of 10-second occlusion is performed, covering the eye that required the longest time to re-fuse. The worse level of control observed following the 3, 10-second periods of occlusion should be recorded for that visit.

13) Distant stereoacuity

- Random Dots Distant Test (P/N 1006, Vision assessment Corporation, Illinois, USA)
- 3 meters
- Ranging from 63 to 400 arcsecs (log stereoacuity values, 1.8 to 2.6 log arcsecs)
- Subjects unable to identify shapes at the 400 arcsecs level are recorded as having ‘nil’ stereo (log stereoacuity value, 3.2 log arcsecs).

14) Near stereoacuity

- Randot Preschool Stereoacuity test (Stereo Optical Co., Inc, Illinois, USA)
- 40 cm
- Testing disparities at 800, 400, 200, 100, 60 and 40 arcsecs
- The result is recorded as the smallest disparity at which the subject could correctly identify at least two of the three shapes.

- Participants who are unable to identify the largest disparity (800 arcsecs) are recorded as having ‘nil’ stereo (log stereoacuity value, 3.2 log arcsecs).

15) Near point of convergence (NPC)

- Near point rule
- The target is gradually moved towards the center of the participant’s eyes at a speed of 1 cm/s until the participant reports double vision, or the examiner notes an outward deviation of one eye.
- The distance from the target to the spectacle plane is recorded as the NPC distance in centimeters.

16) Fusional vergence amplitude (FVA)

- Synoptophore (Haag Streit UK Ltd, Essex, UK)
- Fusion slides subtending a visual angle of 6 degrees horizontally
- The participants are first told to move the arms of the synoptophore by themselves to overlap and fuse the images of a butterfly and a cat. The examiner then slowly abducts or adducts the synoptophore tubes, until 2 distinct images are observed by the participant. The range between the 2 breakpoints

17) Accommodative convergence/accommodation (AC/A) ratio

- Far gradient method
- 6 meters
- Minus lenses (-3.00 D)
- Stimulating a change in accommodation using minus lenses, and dividing the resulting change in deviation (in PDs) by the change in lens power.

18) Wavefront aberrometry

- ITrace (Tracey Technologies, Texas, USA)

19) Choroidal structural and vascular component

Optical coherence tomography (OCT) and optical coherence tomography angiography (OCTA) (Optovue, Inc., Freemont, CA)

20) Cycloplegic autorefraction

- Topcon KR-8900 (Topcon, Tokyo, Japan)
- Instill 6 drops of 1% tropicamide at a 5-minute interval and measure after another 10-minute rest.
- Further cycloplegic eyedrops should be given if the pupillary light reflex is still present or the pupil size is less than 6.0 mm ten minutes after the last eyedrop.

All the procedures will be performed by trained ophthalmologists and optometrists, who are masked for the treatment assignment.

At baseline, the participant will be prescribed best-corrected spectacles if the ones worn are inappropriate for either a difference of spherical power or cylinder power exceeding 0.25D. Participants will be inquired whether photophobia exists at baseline, because some IXT patients may have the symptom.[16]

Daily outdoor hours and self-report symptoms related to allergy, blurred near vision, photophobia, or any discomfort are inquired at each visit.

During the follow-up, if a participant has non-serious ocular events e.g., bacterial or allergic conjunctivitis, the guardian should contact the investigator and stop using eyedrops under the investigator’s guidance. This duration of

stopping treatment should not exceed 4 weeks (28 days).

6. Data collection and management

Before the trial begins, investigators and study coordinators will receive training on trial processes and procedures, including the overview of the trial, consent process, study procedures, ocular evaluation methods, completion of the case report form (CRF), emergency processing and adverse events reporting. The study coordinators will collect all data from trial participants and record it on the paper CRF. Electronic records will be double entered by two investigators separately, and raw data will be checked for inconsistency.

7. Statistical analysis

Statistical analysis will follow intention to treat. Data from children who participate in at least 1 follow-up visit will be included in the analysis. All statistical analyses will be performed using SPSS (IBM Corp, NY, USA) and R (R Foundation for Statistical Computing, https://www.R-project.org/). A two-sided *P*-value <0.05 will be considered statistically significant.

Eye-specific outcome measures will be compared between treatment groups using generalized linear models and generalized estimating equations (GEE) will be used to account for inter-eye correlation. Person-level categorical outcomes will be compared between two treatment groups using chi-square test. For comparing the longitudinal changes of outcome measures

between two treatment groups, GEE models with an autoregressive correlation structure will be used. This longitudinal analysis using GEE will include data from all participants including those who withdraw from the trial during the follow-up.

For the change in SE, AL, and choroidal measures, subgroup analyses will be performed for age (≤8 years vs. >8 years), sex (male vs. female), baseline SE (≤-2 D vs. >-2 D), baseline AL (≤24 mm vs. >24 mm), distant magnitude of exodeviation at baseline (≤ 20 PD vs. >20 PD) and daily outdoor hours (≤2 hours vs. >2 hours). The differences in treatment effect between subgroups will be evaluated based on the test of interaction between treatment group and subgroup.

8. Monitoring

Throughout the trial, the quality control personnel will make routine visits to check procedures of administration and operation, adherence to the protocol, consistency of CRF entries between paper and electronic documents, and compliance with principles of Good Clinical Practice (GCP). The subjects' source documents will be reviewed in detail.

9. Adverse event

An adverse event is defined as any untoward medical occurrence associated with the use of a drug in humans, whether or not the event is considered

drug-related. A serious adverse event is adverse event that led to a death or a serious deterioration in health. Any serious adverse event that occurs must be reported within 24 hours of its occurrence or within 24 hours of learning of its occurrence. Any pre-existing medical condition that worsens after administration of the study drug will also be considered a new adverse event. Documentation regarding the adverse event should include the nature, date of onset, end date, severity, relationship to the study drug, actions taken, seriousness, and outcome of any sign or symptom observed by the physician or reported by the subject upon indirect questioning. Adverse events related to study intervention will be reported to participants through a formal letter sent either physically or using an electronic platform.

The relationship of each adverse event to the study drug should be determined by the investigator using the following explanations:

- Suspected: There is a reasonable possibility that the study drug causes the adverse event.
- Not suspected: There is no reasonable possibility that the study drug causes the adverse event.

10. Ethics and governance

This protocol and the informed consent document and any subsequent modifications will be reviewed and approved by the Chinese Clinical Trial Registry and the institutional review board in the First Affiliated Hospital with

Nanjing Medical University. Informed consent will be obtained from parents or guardians, and additional informed consent will be obtained from participants with age over 8 years old. The consent form will describe the purpose of this study, the procedures to be followed, and the risks and benefits of participating this study.

11. Participant confidentiality

To ensure the privacy of personal information, all study-related data will be stored with access only to the authorized study personnel. Electronic databases will have password-protected access systems. Records containing both names and personal ID numbers will be stored separately and accessible only to the principal investigator and co-investigator.

12. Data access and dissemination policy

It is anticipated that the study findings will be published in national and international peer-reviewed journals and presented at national and international conferences, which will also be disseminated to all study participants and their parents/guardians who wish to be informed. The principal investigator and co-investigator will lead the publications and make the results readily accessible to the public, healthcare professionals, and scientists.

13. Declaration of interests

Any conflicts of interest from individuals involved in the design, implementation, analysis, and publication will be disclosed and managed.

References

1. Govindan M, Mohney BG, Diehl NN, Burke JP. Incidence and types of childhood exotropia: a population-based study. *Ophthalmology*. 2005;112(1):104-8.
2. Pan CW, Zhu H, Yu JJ, et al. Epidemiology of Intermittent Exotropia in Preschool Children in China. *Optom Vis Sci*. 2016;93(1):57-62.
3. Robaei D, Kifley A, Mitchell P. Factors associated with a previous diagnosis of strabismus in a population-based sample of 12-year-old Australian children. *Am J Ophthalmol*. 2006;142(6):1085-8.
4. Cotter SA, Varma R, Tarczy-Hornoch K, et al. Risk factors associated with childhood strabismus: the multi-ethnic pediatric eye disease and Baltimore pediatric eye disease studies. *Ophthalmology*. 2011;118(11):2251-61.
5. C DF. An Essay on the Nature and the Consequences of Anomalies of Refraction *Philadelphia: P Blakiston's Son and Co*. 1899:59.
6. Tang SM, Chan RY, Bin Lin S, et al. Refractive Errors and Concomitant Strabismus: A Systematic Review and Meta-analysis. *Sci Rep*. 2016;6:35177.
7. Ekdawi NS, Nusz KJ, Diehl NN, Mohney BG. The development of myopia among children with intermittent exotropia. *Am J Ophthalmol*. 2010;149(3):503-7.
8. Ahn SJ, Yang HK, Hwang JM. Binocular visual acuity in intermittent exotropia: role of accommodative convergence. *Am J Ophthalmol*. 2012;154(6):981-986 e3.

9. Horwood AM, Riddell PM. Evidence that convergence rather than accommodation controls intermittent distance exotropia. *Acta Ophthalmol*. 2012;90(2):e109-17.

10. Mohney BG, Cotter SA, Chandler DL, et al. Three-Year Observation of Children 3 to 10 Years of Age with Untreated Intermittent Exotropia. *Ophthalmology*. 2019;126(9):1249-1260.

11. Wu PC, Chuang MN, Choi J, et al. Update in myopia and treatment strategy of atropine use in myopia control. *Eye (Lond)*. 2019;33(1):3-13.

12. Chia A, Chua WH, Cheung YB, et al. Atropine for the treatment of childhood myopia: safety and efficacy of 0.5%, 0.1%, and 0.01% doses (Atropine for the Treatment of Myopia 2). *Ophthalmology*. 2012;119(2):347-54.

13. Chia A, Lu QS, Tan D. Five-Year Clinical Trial on Atropine for the Treatment of Myopia 2: Myopia Control with Atropine 0.01% Eyedrops. *Ophthalmology*. 2016;123(2):391-399.

14. Yam JC, Jiang Y, Tang SM, et al. Low-Concentration Atropine for Myopia Progression (LAMP) Study: A Randomized, Double-Blinded, Placebo-Controlled Trial of 0.05%, 0.025%, and 0.01% Atropine Eye Drops in Myopia Control. *Ophthalmology*. 2019;126(1):113-124.

15. Mohney BG, Holmes JM. An office-based scale for assessing control in intermittent exotropia. *Strabismus*. 2006;14(3):147-50.

16. Oh BL, Suh SY, Choung HK, Kim SJ. Squinting and photophobia in intermittent exotropia. *Optom Vis Sci*. 2014;91(5):533-9.
