# Supplementary material for: 0.01% Atropine Eye Drops in Children With Myopia and Intermittent Exotropia: The AMIXT Randomized Clinical Trial
Source: JAMA Ophthalmol. 2024 Jul 3;142(8):722–30. doi: 10.1001/jamaophthalmol.2024.2295 (PMC11223046; doi:10.1001/jamaophthalmol.2024.2295)
Supplement: Supplement 2. — eTable 1. Baseline Characteristics in Participants With and Without Completion of the 12-Month Follow-Up Visit eTable 2. Deterioration Definitions and Outcomes eFigure 1. Frequency for Levels of Change From Baseline in Spherical Equivalent at 1 Year by Treatment Groups eFigure 2. Subgroup Analyses for Change in Axial Length at 1 Year eFigure 3. Change in Monocular Function by Treatment Groups Over Time Including Best-Corrected Visual Acuity, Near Vision, Accommodative Amplitude, Photopic Pupil Size, and Mesopic Pupil Size eFigure 4. Change in Exotropia Conditions by Treatment Groups Over Time Including Distant Magnitude of Exodeviation, Near Magnitude of Exodeviation, Distant Exotropia Control, and Near Exotropia Control eFigure 5. Means and Standard Deviations of Exotropia Conditions by Treatment Groups Over Time Including Distant Magnitude of Exodeviation, Near Magnitude of Exodeviation, Distant Exotropia Control, and Near Exotropia Control eFigure 6. Change in Binocular Vision by Treatment Groups Over Time Including Distant Stereoacuity, Near Stereoacuity, Near Point of Convergence, Accommodative Convergence/Accommodation, Fusional Vergence Amplitude, Fusional Convergence, and Fusional Divergence eFigure 7. Change in Safety Measures by Treatment Group Over Time Including Intraocular Pressure and Corneal Endothelial Cell Density [file jamaophthalmol-e242295-s002.pdf]

Supplementary Online Content

Wang Z, Li T, Zuo X, et al. 0.01% Atropine eye drops in children with myopia and intermittent exotropia: the AMIXT randomized clinical trial. *JAMA Ophthalmol*. Published online July 3, 2024.
doi:10.1001/jamaophthalmol.2024.2295

eTable 1. Baseline Characteristics in Participants With and Without Completion of the 12-Month Follow-Up Visit

eTable 2. Deterioration Definitions and Outcomes

eFigure 1. Frequency for Levels of Change From Baseline in Spherical Equivalent at 1 Year by Treatment Groups

eFigure 2. Subgroup Analyses for Change in Axial Length at 1 Year

eFigure 3. Change in Monocular Function by Treatment Groups Over Time Including Best-Corrected Visual Acuity, Near Vision, Accommodative Amplitude, Photopic Pupil Size, and Mesopic Pupil Size

eFigure 4. Change in Exotropia Conditions by Treatment Groups Over Time Including Distant Magnitude of Exodeviation, Near Magnitude of Exodeviation, Distant Exotropia Control, and Near Exotropia Control

eFigure 5. Means and Standard Deviations of Exotropia Conditions by Treatment Groups Over Time Including Distant Magnitude of Exodeviation, Near Magnitude of Exodeviation, Distant Exotropia Control, and Near Exotropia Control

eFigure 6. Change in Binocular Vision by Treatment Groups Over Time Including Distant Stereoacuity, Near Stereoacuity, Near Point of Convergence, Accommodative Convergence/Accommodation, Fusional Vergence Amplitude, Fusional Convergence, and Fusional Divergence

eFigure 7. Change in Safety Measures by Treatment Group Over Time Including Intraocular Pressure and Corneal Endothelial Cell Density

This supplementary material has been provided by the authors to give readers additional information about their work.

© 2024 Wang Z et al. *JAMA Ophthalmology.*

eTable 1. Baseline characteristics in participants with and without completion of the 12-month follow-up visit.

**Characteristics**	**12-month visit completed**	**12-month visit not completed**	***P* value**
	(N=247)	(N=53)	
Age (years)	9.11 (1.54)	9.20 (1.85)	.73
Sex			.34
Male	122 (49.4)	30 (56.6)	
Female	125 (50.6)	23 (43.4)	
Cycloplegic spherical equivalent (D)	-2.27 (1.69)	-2.45 (1.95)	.37
Axial length (mm)	24.51 (1.16)	24.61 (1.22)	.46
Distant best-corrected visual acuity (logMAR)	-0.05 (0.08)	-0.07 (0.06)	.07
Near vision (logMAR)	0.08 (0.12)	0.08 (0.12)	.76
Accommodative amplitude (D)	17.05 (7.03)	17.44 (7.55)	.62
Photopic pupil size (mm)	2.52 (0.99)	2.42 (0.85)	.27
Mesopic pupil size (mm)	6.36 (1.14)	6.41 (1.00)	.62
Distant magnitude of exodeviation (PD)	20.94 (6.95)	20.83 (7.50)	.92
Near magnitude of exodeviation (PD)	26.84 (7.52)	27.15 (7.05)	.77
Distant exotropia control	2.20 (0.60)	2.26 (0.65)	.53
Near exotropia control	0.66 (0.91)	0.85 (1.04)	.21
Distant stereoacuity (log arcsecs)	3.08 (0.25)	3.12 (0.23)	.28
Near stereoacuity (log arcsecs)	1.71 (0.20)	1.72 (0.20)	.67
Near point of convergence (cm)	4.27 (2.10)	4.35 (2.56)	.84
Accommodative convergence/accommodation	1.81 (1.35)	1.76 (1.12)	.80
Fusional vergence amplitude (°)	19.71 (8.73)	17.69 (8.30)	.11
Intraocular pressure (mmHg)	16.03 (3.71)	16.05 (3.34)	.96
Endothelial cell density (cells/mm^2)	3418.56 (348.82)	3408.74 (273.87)	.75

Abbreviations: D, diopter; PD, prism diopter; Continuous variables are presented as mean (SD); categorical variables are presented as proportion [n (%)].

© 2024 Wang Z et al. *JAMA Ophthalmology.*

eTable 2. Deterioration definitions and outcomes.

Deterioration	0.01% Atropine								Placebo						
	baseline	2m	4m	6m	9m	12m	Cumulative N		baseline	2m	4m	6m	9m	12m	Cumulative N
N at risk	200	200	197	195	191	180	-		100	100	95	92	86	84	-
N with motor deterioration	0	0	0	0	0	0	0		0	0	0	0	0	0	0
[a]N with stereoacuity deterioration	-	2	1	0	0	1	4		-	1	0	1	0	0	2
[a]Cumulative N with motor or stereoacuity deterioration	-	2	3	3	3	4	4		-	1	1	2	2	2	2

Abbreviation: m, months.

Intermittent exotropia was defined as deterioration if any of the following two criteria was met at any follow-up visit after randomization.

Motor criteria: Constant exotropia ≥10 prism diopters at distance and near (throughout exam) by Simultaneous Prism and Cover Test, confirmed by a retest.

Stereoacuity criteria: Drop in near stereoacuity by Randot Preschool Stereotest of at least 2 octaves (0.6 log arcsec) from best stereoacuity of any previous study visit, confirmed by a retest.

[a]All participants with deterioration recovered at the following visit.

© 2024 Wang Z et al. *JAMA Ophthalmology.*

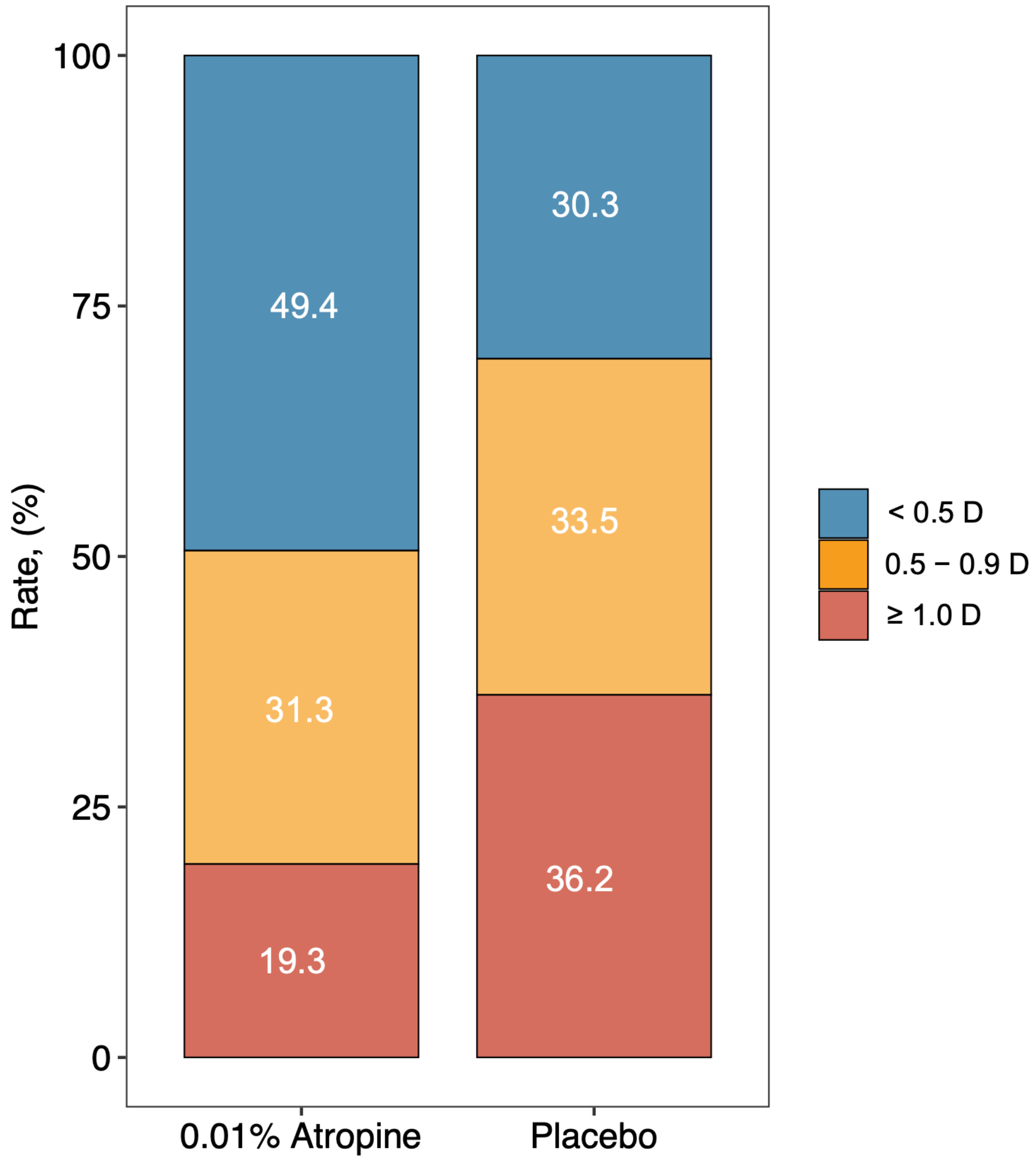

eFigure 1. Frequency for levels of change from baseline in spherical equivalent at 1 year by treatment groups.

© 2024 Wang Z et al. *JAMA Ophthalmology.*

Subgroups	0.01% atropine	N (eyes)	Placebo	N (eyes)	Mean difference (95% CI)	P for interaction
**Age (years)**						**.28**
≤8	0.43 (0.35)	104	0.55 (0.38)	54	-0.13 (-0.25, -0.01)	
>8	0.27 (0.25)	296	0.38 (0.32)	146	-0.11 (-0.17, -0.05)	
**Sex**						**.09**
Male	0.26 (0.24)	204	0.45 (0.39)	100	-0.19 (-0.28, -0.11)	
Female	0.36 (0.32)	196	0.39 (0.32)	100	-0.04 (-0.11, 0.04)	
**Spherical equivalent (D)**						**.06**
≤-2	0.28 (0.32)	285	0.35 (0.35)	135	-0.07 (-0.14, -0.004)	
>-2	0.33 (0.22)	115	0.48 (0.27)	65	-0.15 (-0.23, -0.07)	
**Axial length (mm)**						**.51**
≤24	0.39 (0.33)	101	0.50 (0.39)	66	-0.11 (-0.22, 0.01)	
>24	0.27 (0.25)	299	0.38 (0.31)	134	-0.11 (-0.17, -0.05)	
**Distant magnitude of deviation (PD)**						**.19**
≤20	0.29 (0.28)	228	0.42 (0.38)	118	-0.13 (-0.21, -0.05)	
>20	0.33 (0.30)	172	0.43 (0.31)	82	-0.10 (-0.18, -0.02)	
**Daily Outdoor time (hours)**						**.30**
≤2	0.29 (0.28)	154	0.42 (0.39)	92	-0.13 (-0.23, -0.04)	
>2	0.32 (0.30)	246	0.41 (0.31)	108	-0.10 (-0.17, -0.03)	
**Overall**	**0.31 (0.19)**	**400**	**0.42 (0.22)**	**200**	**-0.11 (-0.17, -0.06)**	

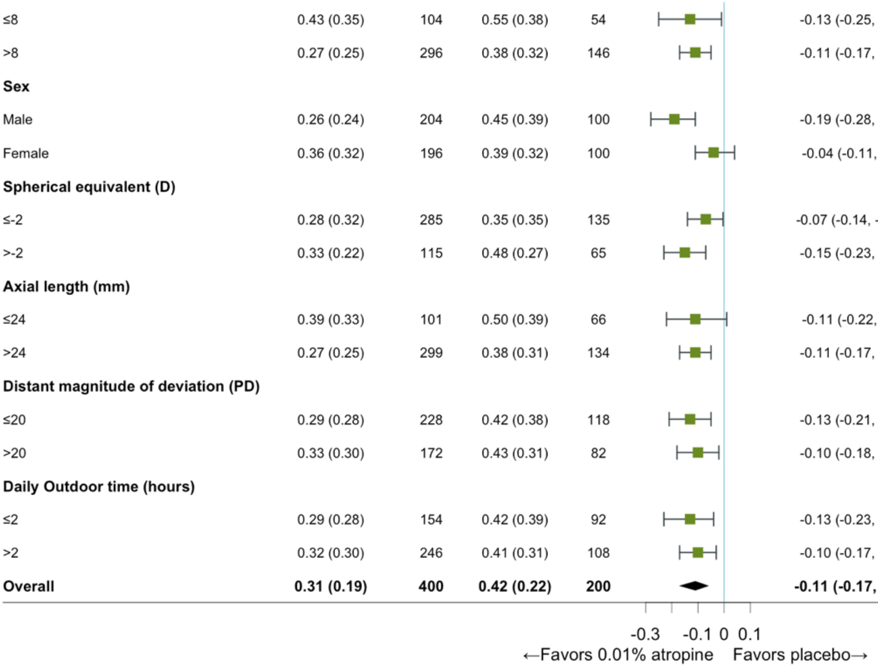

eFigure 2. Subgroup analyses for change in axial length at 1 year. Data are presented with mean and standard deviation. The interaction between treatment groups and subgroups was evaluated using multivariable generalized estimating equations models. Abbreviations: D, diopter; PD, prism diopter; CI, confidence interval.

© 2024 Wang Z et al. *JAMA Ophthalmology.*

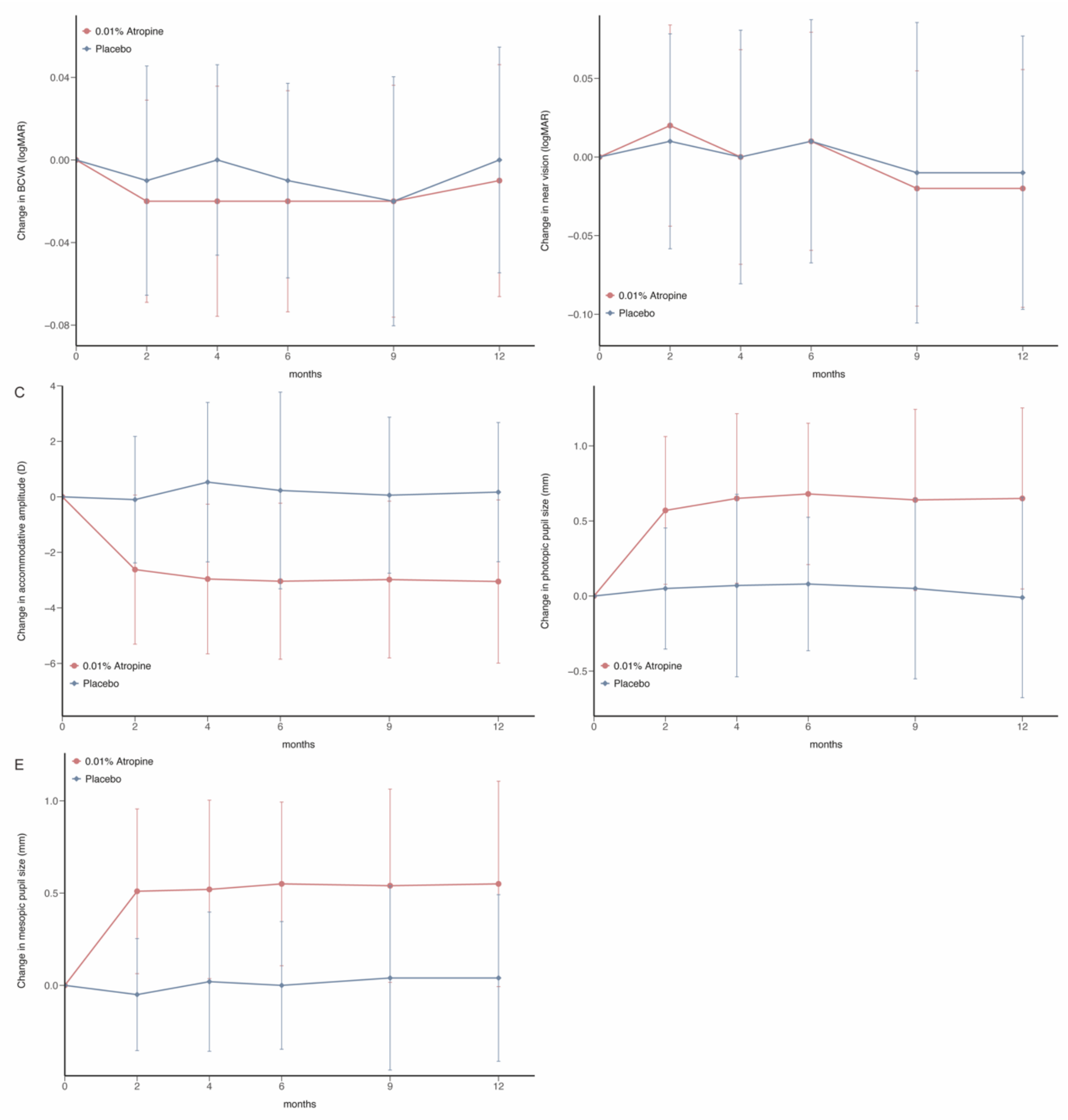

eFigure 3. Change in monocular function by treatment groups over time including best-corrected visual acuity (A), near vision (B), accommodative amplitude (C), photopic pupil size (D) and mesopic pupil size (E). Data are presented with mean and standard deviation over 1 year. Abbreviations: BCVA, distant best-corrected visual acuity; D, diopter.

© 2024 Wang Z et al. *JAMA Ophthalmology.*

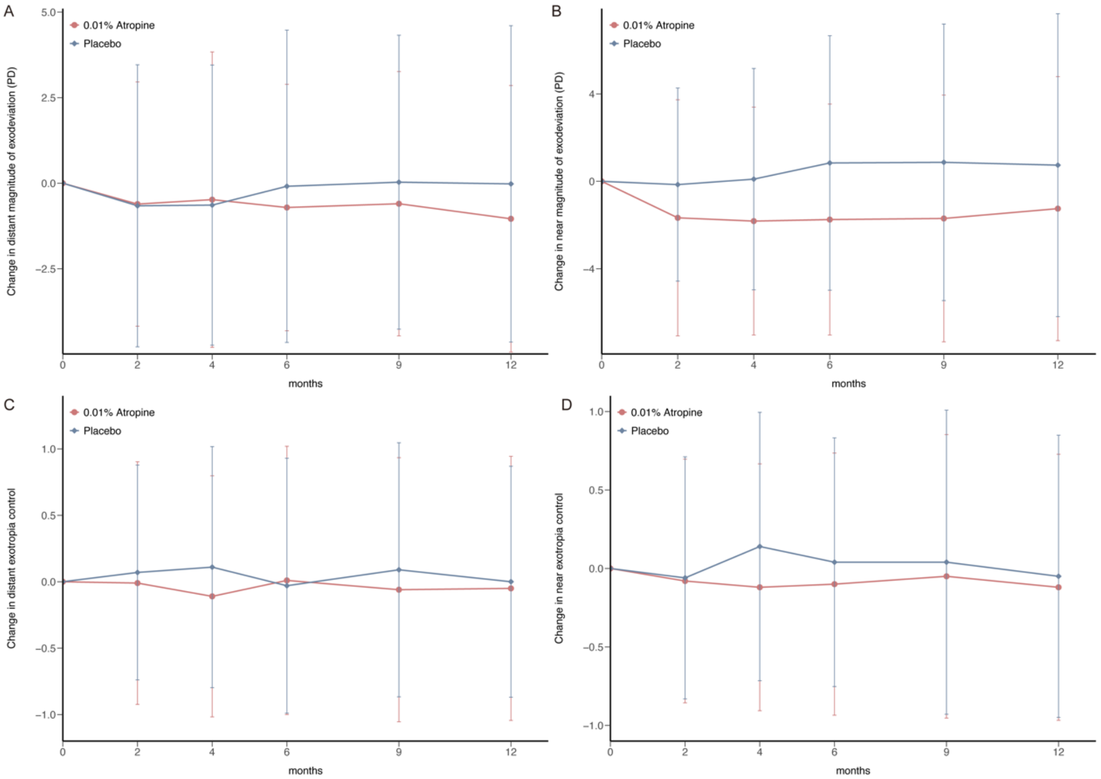

eFigure 4. Change in exotropia conditions by treatment groups over time including distant magnitude of exodeviation (A), near magnitude of exodeviation (B), distant exotropia control (C) and near exotropia control (D). Data are presented with mean and standard deviation over 1 year. Abbreviation: PD, prism diopter.

© 2024 Wang Z et al. *JAMA Ophthalmology.*

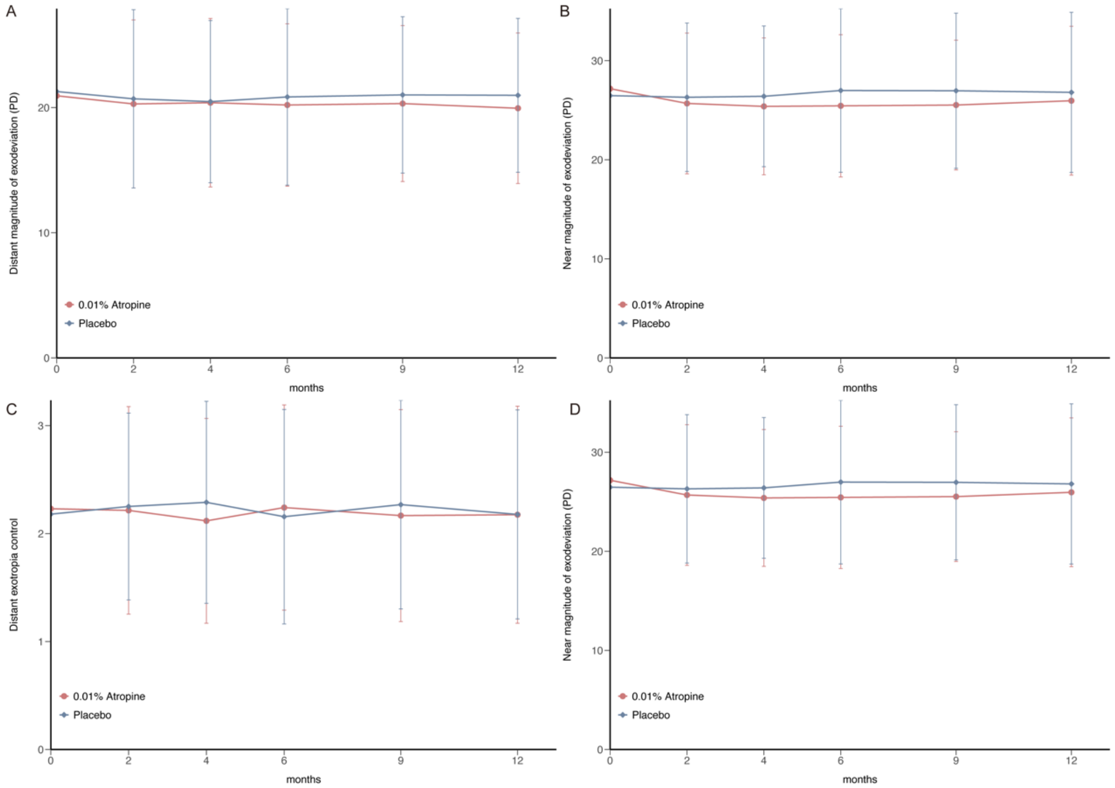

eFigure 5. Means and standard deviations of exotropia conditions by treatment groups over time including distant magnitude of exodeviation (A), near magnitude of exodeviation (B), distant exotropia control (C) and near exotropia control (D). Data are presented with mean and standard deviation over 1 year. Abbreviation: PD, prism diopter.

© 2024 Wang Z et al. *JAMA Ophthalmology.*

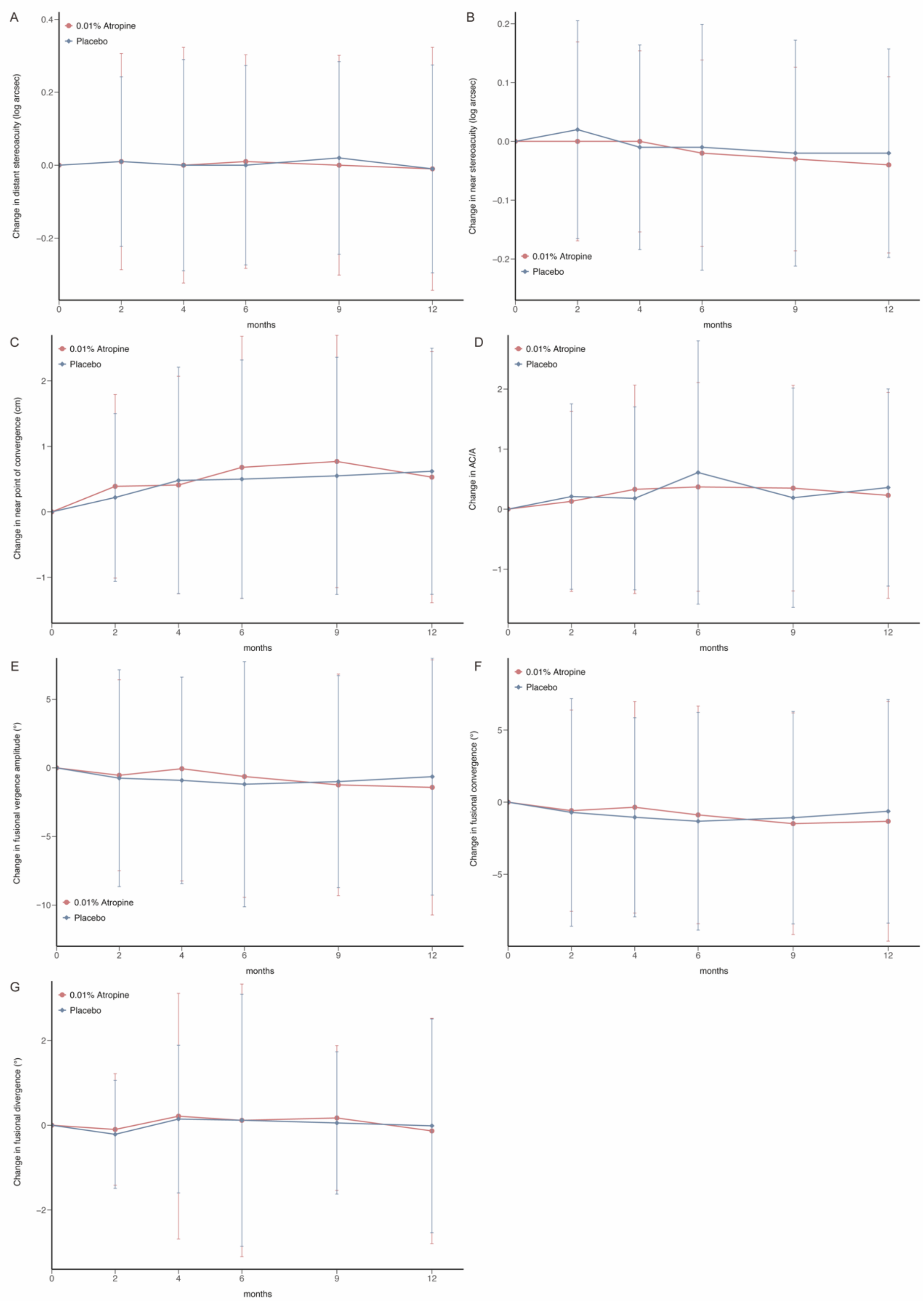

eFigure 6. Change in binocular vision by treatment groups over time including distant stereoacuity (A), near stereoacuity (B), near point of convergence (C), accommodative convergence/accommodation (D), fusional vergence amplitude (E), fusional convergence (F) and fusional divergence (G). Data are presented with mean and standard deviation over 1 year. Abbreviations: AC/A, accommodative convergence/accommodation.

© 2024 Wang Z et al. *JAMA Ophthalmology.*

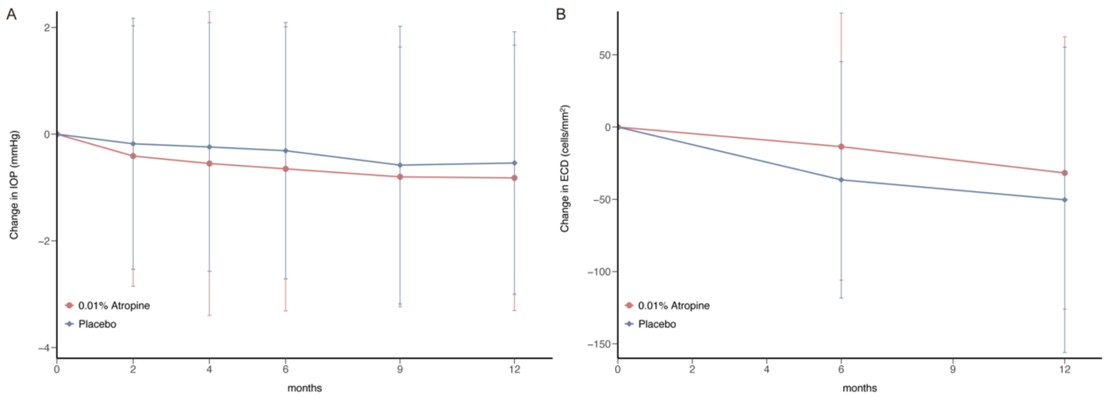

eFigure 7. Change in safety measures by treatment group over time including intraocular pressure (A) and corneal endothelial cell density (B). Data are presented with mean and standard deviation over 1 year. Abbreviations: IOP, intraocular pressure; ECD, corneal endothelial cell density.

© 2024 Wang Z et al. *JAMA Ophthalmology.*
