# Supplementary material for: 0.01% Atropine Eye Drops in Children With Myopia and Intermittent Exotropia: The AMIXT Randomized Clinical Trial
Source: JAMA Ophthalmol. 2024 Jul 3;142(8):722–30. doi: 10.1001/jamaophthalmol.2024.2295 (PMC11223046; doi:10.1001/jamaophthalmol.2024.2295)
Supplement: Supplement 3. — Data Sharing Statement. [file jamaophthalmol-e242295-s003.pdf]

Data Sharing Statement

Wang. 0.01% Atropine Eye Drops in Children With Myopia and Intermittent Exotropia. *JAMA Ophthalmol*. Published July 03, 2024. doi:10.1001/jamaophthalmol.2024.2295

Data
Data available: No

Additional Information
Explanation for why data not available: The data are available from the corresponding author upon reasonable request.
